# Identification of four functionally important microRNA families with contrasting differential expression profiles between drought-tolerant and susceptible rice leaf at vegetative stage

**DOI:** 10.1186/s12864-015-1851-3

**Published:** 2015-09-15

**Authors:** Boon Huat Cheah, Kalaivani Nadarajah, Mayur Dashrath Divate, Ratnam Wickneswari

**Affiliations:** School of Biotechnology and Bioscience, Faculty of Science and Technology, Universiti Kebangsaan Malaysia, 43600 Bangi, Selangor Malaysia; School of Environmental and Natural Resource Sciences, Faculty of Science and Technology, Universiti Kebangsaan Malaysia, 43600 Bangi, Selangor Malaysia; Bionivid Technology [P] Ltd., 401-4AB Cross, 1st Main, NGEF East Kasturi Nagar, Bangalore, 560043 India

## Abstract

**Background:**

Developing drought-tolerant rice varieties with higher yield under water stressed conditions provides a viable solution to serious yield-reduction impact of drought. Understanding the molecular regulation of this polygenic trait is crucial for the eventual success of rice molecular breeding programmes. microRNAs have received tremendous attention recently due to its importance in negative regulation. In plants, apart from regulating developmental and physiological processes, microRNAs have also been associated with different biotic and abiotic stresses. Hence here we chose to analyze the differential expression profiles of microRNAs in three drought treated rice varieties: Vandana (drought-tolerant), Aday Sel (drought-tolerant) and IR64 (drought-susceptible) in greenhouse conditions via high-throughput sequencing.

**Results:**

Twenty-six novel microRNA candidates involved in the regulation of diverse biological processes were identified based on the detection of miRNA*. Out of their 110 predicted targets, we confirmed 16 targets from 5 novel microRNA candidates. In the differential expression analysis, mature microRNA members from 49 families of known *Oryza sativa* microRNA were differentially expressed in leaf and stem respectively with over 28 families having at least a similar mature microRNA member commonly found to be differentially expressed between both tissues. Via the sequence profiling data of leaf samples, we identified osa-miR397a/b, osa-miR398b, osa-miR408-5p and osa-miR528-5p as being down-regulated in two drought-tolerant rice varieties and up-regulated in the drought-susceptible variety. These microRNAs are known to be involved in regulating starch metabolism, antioxidant defence, respiration and photosynthesis. A wide range of biological processes were found to be regulated by the target genes of all the identified differentially expressed microRNAs between both tissues, namely root development (5.3–5.7 %), cell transport (13.2–18.4 %), response to stress (10.5–11.3 %), lignin catabolic process (3.8–5.3 %), metabolic processes (32.1–39.5 %), oxidation-reduction process (9.4–13.2 %) and DNA replication (5.7–7.9 %). The predicted target genes of osa-miR166e-3p, osa-miR166h-5p*, osa-miR169r-3p* and osa-miR397a/b were found to be annotated to several of the aforementioned biological processes.

**Conclusions:**

The experimental design of this study, which features rice varieties with different drought tolerance and tissue specificity (leaf and stem), has provided new microRNA profiling information. The potentially regulatory importance of the microRNA genes mentioned above and their target genes would require further functional analyses.

**Electronic supplementary material:**

The online version of this article (doi:10.1186/s12864-015-1851-3) contains supplementary material, which is available to authorized users.

## Background

To meet the increasing food demand from growing human population in Asia, rice production from different rice growing ecosystems has to be increased as rice is the staple food in this region. However, with the grain yield in irrigated areas reaching stagnation, a large portion of the predicted increase has to come from the water-short drought-prone rainfed lowland and upland rice areas [1]. In Asia alone, about 34 million ha of rainfed lowland and 8 million ha of rainfed upland rice experience drought stress of varying intensities at different stages of the crop almost every year [2]. Moreover, most of the rice varieties presently cultivated in rainfed areas are developed for irrigated conditions. These varieties are highly popular among farmers because of their high yield potential and good grain quality but are highly susceptible to drought, causing substantial yield losses during years of drought [1]. Therefore, due to the increasing threat from water shortage and drought in many rice-growing areas of Asia, particularly the rainfed areas, the development of drought-tolerant varieties with increased yield under drought is a priority area of rice research for sustainable rice production [3]. In Malaysia, aerobic rice farming has been identified as a key area of rice research since 2005 in a bid to safeguard national food security and conserve water [4]. To ensure the success of these breeding programmes, understanding the molecular regulation of this polygenic trait in rice is crucial.

Plants perceive stress signals and respond by reprogramming their gene expression, which enables them to adapt to stress conditions. At the transcriptional regulation level, many stress-responsive genes have been identified using microarray analysis in various plant species, such as *Arabidopsis* [5] and rice [6]. The first group includes molecules such as chaperones, late embryogenesis abundant proteins, osmotin, antifreeze proteins, mRNA-binding proteins, key enzymes for osmolyte biosynthesis, water channel proteins, sugar and proline transporters, detoxification enzymes and various proteases. The second group comprises of regulatory proteins, which include various transcription factors, protein kinases, protein phosphatises, enzymes involved in phospholipid metabolism and other signaling molecules such as calmodulin-binding proteins [7]. A meta-analysis of rice grain yield quantitative trait loci (QTL) identified under drought stress reported seven meta-QTLs with small genetic and physical intervals that could be useful in marker-assisted selection/pyramiding [8]. Three groups of genes were found to occur together in these QTL regions: the stress-inducible genes, growth and development-related genes and sugar transport-related genes [8]. Functional characterization of several of these identified candidate genes have been analyzed via gene transfer, which demonstrated enhanced abiotic stress tolerance in transgenic plants [9–11]. With the discovery of small RNAs, increased attention has been focused on the importance of post-transcriptional gene regulation by microRNAs (miRNAs) during stress.

miRNAs are a large class of small non-coding RNAs that are 21–24 nucleotides (nt) in length. They negatively regulate gene expression at the post-transcriptional level in a wide variety of eukaryotic organisms [12]. miRNAs are present in gene families where each plant miRNA family contains 1–32 loci within a single genome, each potentially encoding identical or nearly identical mature miRNAs [13]. In plants, there are ~20 miRNA families that are well conserved between dicotyledons and monocotyledons. Of these, 7 miRNA families, namely miR156/157, miR160, miR159, miR319, miR165/166, miR390 and miR408 were found in primitive land plants such as *Physcomitrella* and *Selaginella* suggesting that they are highly conserved over wide evolutionary distance [14]. In addition, *Arabidopsis*, rice, *Populus* and *Physcomitrella* possess many non-conserved lineage- or plant species-specific miRNA families [14] and it has been reported that these miRNAs could be of more recent evolutionary origins that arise from duplications of target gene segments [13]. Besides regulating developmental and physiological processes, miRNAs are also associated with different biotic and abiotic stresses as well as to nutrient deficiency in *Arabidopsis*.

Recent studies have reported drought-responsive miRNAs in *Arabidopsis* [15–17], rice [18, 19], maize [20], barley [21], wheat [22], soybean [23], *Populus trichocarpa* [24], *Medicago truncatula* [25] and *Phaseolus vulgaris* [26]. In rice, Zhao et al. [18] initiated the study in identifying drought-responsive miRNAs where oligonucleotide microarray was used to monitor miRNA expressed in rice seedlings under Polyethylene Glycol 6000 induced drought stress. Their study identified miR169g [miRBase:MIMAT0001052] and miR393 [miRBase:MIMAT0000957], as two drought-induced miRNAs [18]. Pyrosequencing data from Sunkar et al. [14] showed that osa-miR1436 [miRBase:MIMAT0005987] and osa-miR1441 [miRBase:MIMAT0005991] were slightly down- and slightly up-regulated respectively by drought stress in rice seedlings. However, their Northern blot studies failed to validate these findings [14]. Until 2010, few stress-related miRNAs were discovered in rice while observations were reported in *Arabidopsis* [27]. However towards the end of 2010, 30 miRNAs were identified as significantly down- or up-regulated in rice under drought stress by microarray analysis where total RNA was extracted from leaf tissue of rice across a wide range of developmental stages, from tillering to inflorescence formation [19]. In another study using the one-tube stem-loop reverse transcription quantitative PCR (ST-RT qPCR) for high-throughput expression profiling of miRNAs in rice seedlings under normal and drought stress conditions, 19 miRNAs were detected to be responsive to drought [27]. However when the results of both these studies were compared, only 5 miRNAs (miR156, miR159, miR169, miR172 and miR408) were found to be commonly regulated by drought stress. The low consistency of findings between both studies may be due to the variation in developmental stage of rice studied, selection of rice varieties, drought treatment imposed, and laboratory analysis methods used.

More miRNAs are being identified through high throughput sequencing and various other methods and this information is being deposited in the rice miRBase database. In studying drought response in rice inflorescence tissue via high-throughput sequencing, Barrera-Figueroa et al. (2012) reported 18 miRNAs that were differentially regulated by drought stress [28]. Hence we believe that there are certainly more drought-responsive miRNAs that may be discovered through comparative analysis of data acquired from drought resistant, tolerant and susceptible lines. Through high-throughput sequencing, we have profiled drought-responsive miRNAs in leaf and stem tissues of vegetative rice plants with different levels of drought tolerance. The high sensitivity, specificity and reproducibility of this next-generation sequencing technology has provided a good means of differentially profiling drought-responsive miRNAs and identifying novel miRNAs, which are difficult to be detected due to their low expression levels. Here we report for the first time drought-responsive miRNA expression profiles in two tissues and in three rice varieties with different drought tolerance. In addition to reporting the miRNA profiling data, we also short-listed differentially expressed miRNAs, which have displayed either an interesting differential expression patterns under drought stress or have been linked to target gene(s) that have been annotated to different important biological processes that are either directly or indirectly related to the regulation of stress in plants.

## Results and discussion

### Overview of sequence pre-processing, length distributions and database mapping

Leaf and stem tissues were harvested at the vegetative stage of three rice varieties with different drought tolerance levels grown under control and drought stress conditions for the construction and sequencing of 12 small RNA libraries. The sequence pre-processing stage (Additional file 1), which includes five quality control steps, processes Solexa 50 nt tags or raw reads into high quality clean reads. Post pre-processing of the raw reads, an average of 16847232, 15403441 and 18622662 clean reads were retrieved for Vandana, Aday Sel and IR64 small RNA libraries respectively (Additional file 2).

Four length distributions of small RNA reads (Additional file 3) from Vandana libraries consistently showed 21 and 24 nt peaks while those of Aday Sel libraries showed 21 nt peak consistently. However, the four length distributions of small RNA reads from IR64 libraries were more variable in terms of peak pattern with control condition leaf library showing 21 and 24 nt peaks, while the control stem library showed 20, 21 and 24 nt peaks. The drought leaf and stem libraries showed peaks at 21 and 20 nt respectively. The observation of 20 nt peak in the length distribution of (drought, stem) IR64 library coincides with the mapping distribution of clean reads to over 60 % of transfer RNAs (tRNAs). A novel class of small RNAs, tRNA-derived RNA fragment (tRFs) was reported by Lee et al. [29]. Nevertheless, we cannot rule out the possibility that the variability of peak pattern in the length distributions from IR64 small RNA libraries and the vast increase of reads that mapped to tRNAs in the (drought, stem) library was caused by the actual biological changes happening in the plant cell system when exposed to drought owing to IR64’s drought susceptibility phenotype and the severity level of the drought treatment imposed. Overall the length distributions of small RNA reads indicate that the libraries are highly enriched in miRNAs in the 21–24 nt lengths.

The results of mapping the clean reads to different publicly available databases (Table 1) showed a consistent percentage of proportions with previous study [30]. In Li et al. [30], their known rice miRNAs account for 17.2–20.5 % of total clean reads with a very small fraction (~0.1 %) of unique clean reads in their small RNA libraries. In our mapping results, known rice miRNA sequences account for 26.5–44.0 % of total clean reads and ~0.2 % of unique clean reads in the small RNA libraries (Table 1). Once again, this mapping results show that our small RNA libraries were highly enriched in miRNAs as compared to the previous report [30]. In addition 59.5–62.4 % of these are unannotated unique reads that may possibly contain novel miRNA candidates and other classes of small RNAs.

**Table 1:** Mapping distribution of the clean reads in small RNA libraries

Categories	Vandana		Aday Sel		IR64	
	Total reads	Unique reads	Total reads	Unique reads	Total reads	Unique reads
Clean reads	16 847 232 ± 1 528 242	3 332 304 ± 635 063	15 403 441 ± 1 282 332	2 394 590 ± 675 288	18 622 662 ± 1 208 043	3 643 737 ± 1 094 125
9311 rice genome	10 249 097 ± 1 228 933 (60.8 %)	1 480 850 ± 296 597 (44.4 %)	10 647 829 ± 1 682 873 (69.1 %)	793 346 ± 194 986 (33.1 %)	13 818 525 ± 2 320 473 (74.2 %)	1 614 944 ± 469 863 (44.3 %)
Exon antisense	123 658 ± 31 951 (0.7 %)	37 913 ± 10 407 (1.1 %)	143 789 ± 58 608 (0.9 %)	29 918 ± 8184 (1.3 %)	135 144 ± 31 018 (0.7 %)	43 449 ± 9513 (1.2 %)
Exon sense	148 224 ± 44 300 (0.9 %)	84 852 ± 23 724 (2.6 %)	159 436 ± 46 424 (1.0 %)	80 885 ± 14 721 (3.4 %)	184 756 ± 32 941 (1.0 %)	85 802 ± 26 065 (2.4 %)
Intron antisense	115 867 ± 30 826 (0.7 %)	50 016 ± 10 263 (1.5 %)	50 944 ± 20 434 (0.3 %)	26 964 ± 7620 (1.1 %)	132 987 ± 40 799 (0.7 %)	55 629 ± 15 460 (1.5 %)
Intron sense	188 761 ± 62 005 (1.1 %)	58 543 ± 12 020 (1.8 %)	83 925 ± 30 603 (0.5 %)	33 330 ± 8440 (1.4 %)	182 440 ± 36 679 (1.0 %)	62 038 ± 15 960 (1.7 %)
Known miRNA	4 457 106 ± 1 511 316 (26.5 %)	6037 ± 490 (0.2 %)	6 769 286 ± 1 144 378 (44.0 %)	5598 ± 985 (0.2 %)	5 404 163 ± 2 597 947 (29.0 %)	5759 ± 556 (0.2 %)
rRNA	1 046 140 ± 299 191 (6.2 %)	77 037 ± 12 331 (2.3 %)	1 785 015 ± 239 128 (11.6 %)	102 438 ± 13 644 (4.3 %)	699 723 ± 182 679 (3.8 %)	65 568 ± 9341 (1.8 %)
Repeat	2 433 147 ± 506 069 (14.4 %)	1 005 986 ± 166 997 (30.2 %)	1 547 941 ± 684 735 (10.1 %)	666 553 ± 189 898 (27.8 %)	2 609 401 ± 865 194 (14.0 %)	1 029 967 ± 288 440 (28.3 %)
snRNA	10 215 ± 4326 (0.1 %)	2250 ± 489 (0.1 %)	17 203 ± 1154 (0.1 %)	3136 ± 391 (0.1 %)	5920 ± 907 (0.0 %)	1566 ± 175 (0.0 %)
snoRNA	18 859 ± 5210 (0.1 %)	3261 ± 677 (0.1 %)	9806 ± 2330 (0.1 %)	2986 ± 598 (0.1 %)	9433 ± 2721 (0.1 %)	2816 ± 263 (0.1 %)
tRNA	2 428 011 ± 1 192 154 (14.4 %)	18 784 ± 3404 (0.6 %)	2 035 604 ± 2 480 765 (13.2 %)	18 371 ± 3104 (0.8 %)	4 904 192 ± 5 445 456 (26.3 %)	17 397 ± 3844 (0.5 %)
Unannotated	5 877 246 ± 1 887 347 (34.9 %)	1 987 625 ± 398 270 (59.7 %)	2 800 494 ± 1 023 410 (18.2 %)	1 424 411 ± 473 219 (59.5 %)	4 354 504 ± 1 328 748 (23.4 %)	2 273 749 ± 737 311 (62.4 %)

In each rice variety, number of reads is shown as the mean of the four libraries generated ± standard deviation. Percent of the total clean reads is shown in bracket

### The expression profile of known rice miRNAs

Rice, as a model species of monocotyledons, has been subject to substantial effort of miRNA discovery. As a result, miRBase (version 21) records 713 mature miRNAs and 592 miRNA precursors for rice. Statistics of mapping the clean reads from our 12 small RNA libraries to the rice miRNA database showed that 444–480 mature miRNAs and 366–397 miRNA precursors had been mapped (Additional file 4) and were consistent in the mapping results of all the small RNA libraries.

Mature miRNA(s) from 21 conserved miRNA families were detected in each of the libraries and their expression level counts were tabulated (Additional file 5A). Our results showed that the expression level of conserved miRNA families can be classified into three categories viz. as high [transcripts per million (TPM) > 10000/100000; osa-MIR168, osa-MIR156, osa-MIR166], moderate (TPM = 100–10000; osa-MIR167, osa-MIR397, osa-MIR408, osa-MIR159, osa-MIR164, osa-MIR172, osa-MIR396) and low (TPM < 100; osa-MIR160, osa-MIR162, osa-MIR169, osa-MIR171, osa-MIR390, osa-MIR393, osa-MIR394, osa-MIR395, osa-MIR398, osa-MIR399, osa-MIR827). For example, MIR168 [miRBase:MIPF0000081], the most highly expressed conserved miRNA family (TPM > 100000) is known to feedback regulate miRNA biosynthesis pathway by cleaving *ARGONAUTE 1* and this regulation is crucial for plant development [31]. However, the low expression of osa-MIR395 [miRBase:MIPF0000016] and osa-MIR399 [miRBase:MIPF0000015] is in agreement with the findings of Mallory et al. [32] that reported these miRNAs as non detectable in plants grown under standard conditions, but were induced by low-sulphate and low-phosphate stresses respectively. Conserved miRNAs recorded higher reads than non-conserved miRNAs in all of the 12 small RNA libraries, with over 85.2–97.7 % of the mapped reads derived from the conserved miRNAs in each library, except in (control, leaf) and (control, stem) Vandana libraries that recorded only ~68.0 %. The lower percentage of conserved miRNA reads in both the Vandana libraries above is caused by the notably higher expression level of osa-miR528-5p [miRBase:MIMAT0002884], a non-conserved miRNA (Additional file 5B). This could be an interesting preliminary finding as higher expression level of osa-miR528-5p was unique to Vandana, a drought-tolerant rice variety in drought-free condition compared to Aday Sel and IR64. osa-miR528-5p was also found to exhibit interesting differential expression patterns and its important molecular function will be discussed in the corresponding section. Out of the 220 rice mature miRNAs in miRBase that were not mapped by the clean reads of any of our libraries, only 18 (8.2 %) are conserved miRNAs. Majority of the non-conserved miRNAs were not mapped by clean reads due to either one of the following reasons: (i) they are expressed at low levels physiologically; (ii) they have tissue specificity (example detected in rice grain, inflorescence, callus or pollen); (iii) they are predicted miRNA homologs identified by Illumina deep sequencing in barley only (osa-miR5071, osa-miR5074, osa-miR5075, osa-miR5077, osa-miR5080 and osa-miR5081); or (iv) they lack experimental proof (osa-413 to osa-miR426).

We observed that some miRNA members are highly expressed while others are lowly expressed in a miRNA family. In this aspect, we compared our expression profile of known rice miRNAs (Additional file 5) with that from rice seedlings subjected to hydrogen peroxide stress analysis, which was also generated by Solexa high-throughput sequencing [30]. Expression profiles from both studies clearly show consistency in identifying the highly expressed and lowly expressed members, especially for 10 conserved miRNA families (MIR156, MIR160, MIR162, MIR164, MIR166, MIR167, MIR168, MIR171, MIR172 and MIR396). For example, osa-MIR168, which is a highly expressed miRNA family, has two miRNA members (osa-miR168a-5p [miRBase:MIMAT0001045] and osa-miR168b [miRBase:MIMAT0001046]). Only osa-miR168a-5p (TPM > 10000/100000) was found to be highly expressed while osa-miR168b was completely undetected. Another highly expressed miRNA family, osa-MIR156 has over 12 miRNA members. We found that osa-miR156a-j (TPM = 10000–100000) were highly expressed while osa-miR156k [miRBase:MIMAT0001020] (TPM < 200) and osa-miR156l-5p [miRBase:MIMAT0001021] (TPM < 10) were lowly expressed. In the lowly expressed osa-MIR171 family that has nine miRNA members, we found that osa-miR171b-f (TPM = 10–100) was expressed at higher levels than osa-miR171a [miRBase:MIMAT0000645], osa-miR171g [miRBase:MIMAT0001068] and osa-miR171h [miRBase:MIMAT0001077] (TPM < 1). However, our study showed a higher expression level for osa-miR171i-3p [miRBase:MIMAT0001085], which is approximate to levels seen in osa-miR171b-f, compared to that of the previous study [30]. This conformity in results implies that the high-throughput sequencing used in this study is specific enough to differentiate the miRNA members that vary by a single nucleotide and even in the lowly expressed miRNA families.

Although each annotated miRNA in miRBase is a single defined sequence with no further details on possible variable sequence length, the population of variants of miRNAs coming from the same precursors (isomiRs), have been identified in several species and could shed more light on the mechanism of action of these tiny regulators [33]. It has also been suggested that isomiRs might play a role in determining the stability/subcellular localization, functional efficacy or miRNA target-specificity [34]. In this study, many mature miRNA variants of the annotated miRNAs in miRBase were observed based on the criterion that a mature miRNA sequence is mostly represented in the 12 small RNA libraries (Additional file 5). Previously reported variants that were observed within a ±2 nt range away from the 5′ or 3′ ends of the annotated miRNAs such as osa-miR156l [miRBase:MIMAT0001021], osa-miR156k [miRBase:MIMAT0001020], osa-miR167d-j, osa-miR319a/b, osa-miR820a-c and osa-miR1432-5p [miRBase:MIMAT0005966] [30] were among the many mature miRNA variants detected in our study (Additional file 5). Our results also confirmed the 3 nt shift of osa-miR171i-3p [miRBase:MIMAT0001085] and osa-miR397a/b reported in previous study [30]. In view of the considerably high number of small RNA libraries sequenced deeply through the next-generation sequencing technology, the miRNA variants detected in this study can be reliably referred to by future studies.

### Identification of 26 novel miRNA candidates

The formation of stable hairpin structure is one of the essential features for identification of new miRNAs [35]. In this study, secondary hairpin structures of the putative miRNA candidates were produced and evaluated using Mireap software. Consequently we identified 275 putative miRNA candidates based on the criteria that they are derived from hairpin precursors with stable secondary structures and were detected in at least two small RNA libraries (Additional file 6A). Lan et al. [36] reported that most novel rice miRNAs showed weak expression levels (sequencing frequency <50), where a majority of the putative miRNA candidates identified in this study (86.5 %) had expression levels <100 and while a few showed expression levels in the order of hundreds (10.2 %) or thousands (3.3 %). Among them, miRNA* were detected for 27 putative miRNA candidates. The detection of miRNA* serves as evidence that they are DICER-LIKE 1 (DCL1) cleavage products [37] and thus are considered to be novel miRNA candidates (Additional file 6B). However, cand_mir_76-5p and cand_mir_281-5p were derived from the same precursor with 1 nt shift in their mature miRNA sequences due to unspecific cleavage of DCL1 protein. Out of the 12 small RNA libraries, cand_mir_76-5p was detected in 10 libraries and cand_mir_281-5p was detected in the other two libraries. Therefore, we identified a total of 26 novel miRNA candidates and their secondary structures are shown in Additional file 7. Among them, the hairpin precursors of 5 novel miRNA candidates, namely cand_mir_76-5p/cand_mir_281-5p, cand_mir_125-5p, cand_mir_124-3p, cand_mir_ 322-3p and cand_mir_296-5p were predicted to be transcribed from both strands of the genome DNA at the same locus indicating that the miRNAs are likely to be derived from both the sense and antisense hairpin precursors. This type of small RNA has previously been reported using bioinformatics analysis [38]. Although we could not confirm whether they are bona fide miRNAs and whether both strands of precursor are functionally important in miRNA biosynthesis from our studies, we however observed 100 % sequence complementarity between miRNA and miRNA* in all 5 of them by inspecting the secondary structures of their precursors (Additional file 7). It is however possible that the high sequence complementarity could result in small RNAs being detected on both precursor strands and does not necessarily mean that both strands are functionally viable. To our best knowledge, all of these 26 miRNA candidates have not been reported thus far in rice or in other species and hence substantiating their novelty.

**Fig. 1:**
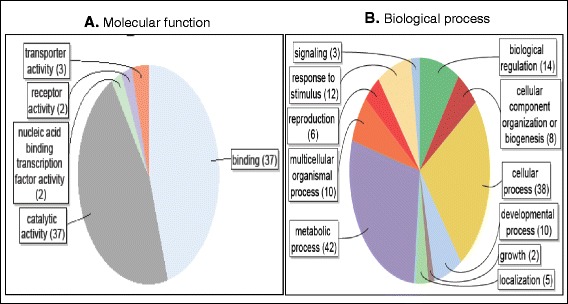
Pie chart showing the distribution of GO annotations of the 26 novel miRNA candidates’ targets. **a** The distribution of annotated GO molecular function. **b** The distribution of annotated GO biological process. Numbers in parentheses are the numbers of annotations in a particular category

Over 110 targets were predicted for the 26 novel miRNA candidates (Additional file 6C) with cand_mir_75-3p having the highest number (21) of predicted targets. cand_mir_327-5p, cand_mir_251-5p and cand-mir_2-5p were respectively predicted to have single targets, with no Gene Ontology (GO) annotation available for these targets. Out of 76 targets annotated with GO molecular functions, over 91.4 % are equally contributed by catalytic activity and binding activity as shown in Fig. 1a. Nucleic acid binding transcription factor activity only consists of 2.5 % (Fig. 1a) and this low percentage clearly shows that these newly identified miRNAs are unlikely conserved miRNA families that target predominantly transcription factors [39]. Under the catalytic activity, there are the transferases (45.7 %) which include glucosyltransferase and phophotransferase activities, the hydrolases (34.3 %) and oxidoreductases (20.0 %). As for the binding activity, it is divided into ion binding (30.4 %), small molecule binding (28.3 %) such as ATP molecule, nucleic acid binding (23.9 %), protein binding (10.9 %) and cofactor binding (6.5 %). The predicted targets were also annotated to diverse biological processes where the metabolic and cellular processes top the list (Fig. 1b). Previously, Tian Li’s group reported that the majority of new miRNAs are predicted to target mRNAs encoding important enzyme functions such as protein kinases, peroxidases and glyoxalases that are widely involved in the regulation of diverse biological processes [30]. All the details of the GO annotation for the predicted targets are shown in Additional file 6C.

Deep sequencing of mRNA cleavage products (degradome sequencing) provides a means for confirmation of miRNA targets [28]. To confirm the 110 predicted miRNA targets of the 26 novel miRNA candidates in this study, we searched the published degradome dataset from *Oryza sativa* ssp. japonica [40] using the CleaveLand software [41]. We were able to confirm 16 targets for 5 novel miRNA candidates (Additional file 6C). For instance, cand_mir_76-5p/c and_mir_281-5p targets putative polygalacturonase, cytokinin-O-glucosyltransferase and exostosin. Polygalacturonase (EC 3.2.1.15) hydrolyzes the α-1,4 glycosidic bonds between galacturonic acids in the pectin network of plant cell wall. Cytokinin-O-glucosyltransferase (EC 2.4.1.203) catalyzes glucosylation of zeatin to form glucosyl zeatin (a cytokinin storage product). Exostosin family consists of glycosyltransferases that involve in the synthesis of Heparan Sulfate backbone, which is essential for cellular signal transduction [42]. Taken together, hexoses are the common substrate residues of these three proteins targeted by cand_mir_76-5p/cand_mir_281-5p. cand_mir_322-3p targets putative protein phosphatase 2C (EC 3.1.3.16) that has been described as negative regulator in the abscisic acid mediated signalling network [43]. cand_mir_75-3p targets putative pectinesterase inhibitor (EC 3.1.1.11; modification of cell walls via demethylesterification of cell wall pectin) and AP2 domain-containing protein (regulation of developmental processes such as floral organ identity) [44]. cand_mir_427-5p targets putative DEAD-box ATP-dependent RNA helicase and glycine rich RNA-binding protein, and cand_mir_388-5p targets putative dihydrodipicolinate reductase (EC 1.3.1.26) that catalyzes the biosynthesis of lysine.

Based on the functional consistency of predicted targets, the functional regulation of some novel miRNA candidates were identified. For example, cand_mir_205-5p was detected in eight small RNA libraries with expression level of <66. It was predicted to target transcripts encoding Armadillo/β-catenin repeat and importin subunit α-1-like. Armadillo repeat is a characteristic repetitive amino acid sequence found in α-importin [45]. Importin, a karyopherin, is involved in the transportation of protein molecules into the nucleus by binding to a specific recognition sequence, called the nuclear localization signal (NLS) [46]. Its involvement in regulating the housekeeping functions of nuclear transport makes it an important component hence justifying cand_mir_205-5p detection in eight of the small RNA libraries. cand_mir_448-5p was also predicted to target transcripts encoding receptor-like protein kinases (Additional file 6C).

The differential expression of the identified 275 putative miRNA candidates has not been reported here due to the low expression of these miRNA candidates that may result in excessive false positive results. However, the expression of these evolutionarily new miRNAs [13] could increase in plants as they become more functionally important. It is worthwhile to highlight a putative miRNA candidate, cand_mir_283-5p, which was only expressed in drought stressed conditions in Vandana and Aday Sel, with low expression levels (26–135). It is predicted to target Auxin response factors (*ARF*) 2 and 4. miR160 [miRBase:MIPF0000032] and miR167 [miRBase:MIPF0000023] are the two conserved miRNA families known to target *ARF*s. Hence, this shows that cand-mir_283-5p could be a new miRNA that may be related to auxin signalling.

### Identification of drought-responsive known miRNAs

**Fig. 2:**
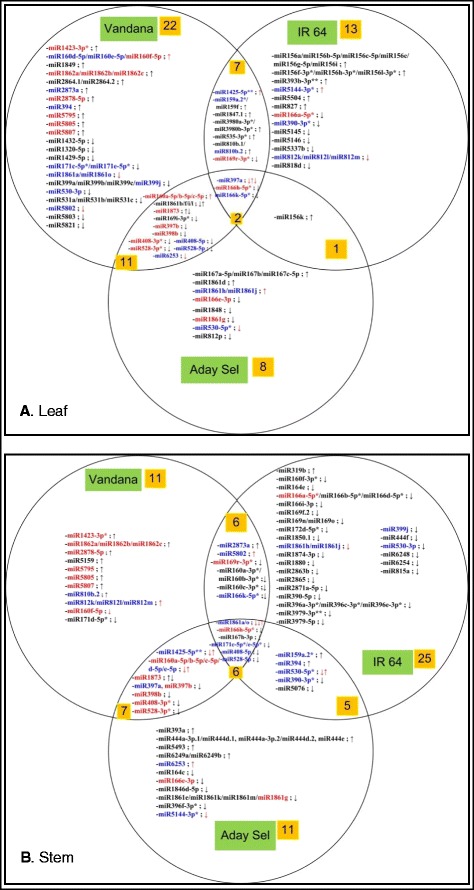
Venn diagram showing the distribution of differentially expressed known miRNAs between 3 rice varieties. **a** The distribution in leaf tissues. **b** The distribution in stem tissues. A * after the miRNA name indicates that it is a miRNA*. A few miRNA names are followed by **, this sign indicates that the miRNA* is having a comparable or higher level of expression compared with mature miRNA. Blue highlighted miRNA names indicate that they are commonly found to be differentially expressed between both tissues. Red highlighted miRNA names indicate that they are commonly found to be differentially expressed in the same rice variety(s) between both tissues. The arrows indicating up-regulation or down-regulation are arranged in the order of Vandana, IR64 and Aday Sel. Red highlighted arrows indicate that they are in opposite direction of differential expression between both tissues

The significant change in expression level of a miRNA observed between drought-treated and control samples may indicate miRNAs that are functionally responsive towards drought stress. A Venn diagram was generated for the miRNAs in the three rice varieties for leaf and stem tissues (Fig. 2). Over 64 and 71 groups of mature miRNAs (including miRNA*) were found to be differentially expressed in leaf and stem respectively, with 28 (43.75 %) and 19 (26.76 %) of these mature miRNAs being up-regulated. Some of the mature miRNAs are grouped together in the Venn diagram because they have identical mature miRNA sequence. miRNA* was included in our differential expression analysis as the accumulation of some rice miRNAs*, but not their corresponding miRNAs, have been reported in Rice stripe virus (RSV) infection [47]. The target prediction analysis for the differentially expressed miRNAs* could be conducted with the same parameter settings used for miRNAs (Additional file 8). One such example of our prediction analysis is osa-miR166h-5p* [miRBase:MIMAT0022882] which was down-regulated in all of our libraries and predicted to target several GO annotated genes such as Diaminopimelate decarboxylase, U-box domain containing protein, and stress-induced protein STI1 (Table 2). Even though the differential expression analysis of miRNA* is more predisposed to false positive errors due to its low expression level, our findings on miRNA* still suggest the potential functional role of miRNA* in the complex miRNA regulatory circuitry (Additional file 8). Interestingly, 39 mature miRNAs were commonly differentially expressed in both tissues, of which 21 mature miRNAs (highlighted in blue) were categorized in different spaces of the Venn diagram and the remaining 18 mature miRNAs (highlighted in red) were categorized within the same space of the Venn diagram (Fig. 2).

**Table 2:** Differentially expressed miRNAs with target(s) that regulate several processes

Mature miRNA	Expression pattern	Target(s)	GO biological process(s)
osa-miR166e-3p	A↓	Alkaline neutral invertase	Root development, carbohydrate metabolic process, cellular amino acid metabolic process
osa-miR166h-5p^a^	V↓R↓A↓	Diaminopimelate decarboxylase	Cellular amino acid metabolic process
		U-box domain containing protein	Protein phosphorylation
		Stress-induced protein STI1	Protein phosphorylation, protein ubiquitination
osa-miR169r-3p^a^	V↓R↓	UDP-glucose 4-epimerase	Root development, response to stress, carbohydrate metabolic process, cell wall biogenesis
osa-miR397a/b	Leaf- V↓R↑A↓	Osmotic stress-activated protein kinase	Response to salt stress, protein phosphorylation
	Stem- V↓A↓	Laccase-22 & laccase lac 5-4	Lignin catbolic process, oxidation-reduction process

^a^after miRNA name indicates that the mature miRNA is miRNA*

*V* Vandana, *A* Aday Sel, *R* IR64

In addition to the Venn diagrams, the drought-responsive known mature miRNAs were also tabulated according to their families, which were categorized into conserved and non-conserved miRNA families (Additional file 8). Information like the differential expression patterns in both tissues and their predicted targets is also summarized in Additional file 8. Our results showed that mature miRNAs from over 49 known miRNA families were differentially expressed in leaf and stem respectively with mature miRNAs from over 28 families being differentially expressed between both tissues.

Among the 21 highly conserved miRNA families in rice, mature miRNAs of over 15 and 16 families were found to be differentially expressed in leaf and stem respectively with 11 families having at least a common mature miRNA member that was differentially expressed between both tissues such as osa-MIR159, osa-MIR160, osa-MIR166, osa-MIR169, osa-MIR171, osa-MIR390, osa-MIR394, osa-MIR397, osa-MIR398, osa-MIR399 and osa-MIR408 (refer to Additional file 8 for their functional information). Another two families (osa-MIR167 and osa-MIR393) were also commonly found to be differentially regulated between both tissues where the members of the family differentially regulated in each tissue were different. We also found that miRNA(s) from osa-MIR156 [miRBase:MIPF0000008] and osa-MIR827 [miRBase:MIPF0000726] was only differentially expressed in leaf while osa-MIR164 [miRBase:MIPF0000045] was differentially expressed in stem. This result indicates the tissue specificity of these miRNA families in modulating drought stress responses in rice. For example, in our analysis, 11 out of 12 mature miRNAs in osa-MIR156 were leaf-specific in their differential expression under drought stress only in IR64. This observation is logical since miR156 is known to negatively regulate Squamosa promoter binding protein (*SPB* or *SPL*) family transcription factors that play an important roles in leaf development and vegetative phase change [48]. The MIR164 family is known to target the No Apical Meristem (*NAM-NAC*) and cup-shaped cotyledon (*CUC*) transcription factor coding genes, which are reported to be important in root and shoot development [49]. Therefore, this explains the stem-specific differential expression of osa-MIR164 family observed in our drought stress experiment.

In addition, we also found that all 6 mature miRNAs from osa-MIR160 family [miRBase:MIPF0000032] were up-regulated in leaf while down-regulated in stem of Vandana rice variety. This implies that the opposite miRNA regulation of osa-MIR160 members is taking place in both tissues when Vandana rice variety is subjected to drought stress. MIR160 family has been confirmed to target *ARF 10*, *ARF16*, and *ARF17* [50]. These ARF transcription factors bind to TGTCTC auxin response elements in promoters of early auxin response genes [51]. This shows the upstream regulatory role of MIR160 family in auxin signal transduction which is important for plant growth. It was reported that plants with miR160-resistant forms of the *ARF10*, *ARF16* and *ARF17* genes showed pleiotropic effects in shoots and roots [49]. Besides that, miR160 family has been reported to play major roles in drought and abscisic acid response in plants [52].

For non-conserved miRNA families, we found over 34 and 33 families were differentially expressed in leaf and stem respectively with 17 families commonly found between both tissues. They are mature miRNAs from osa-MIR1423, osa-MIR1425, osa-MIR1861, osa-MIR1862, osa-MIR1873, osa-MIR2873, osa-MIR2878, osa-MIR5144, osa-MIR528, osa-MIR530, osa-MIR5795, osa-MIR5802, osa-MIR5805, osa-MIR5807, osa-MIR6253, osa-MIR810 and osa-MIR812 families. Additional file 8 shows that all 12 mature miRNAs of osa-MIR1861 family [miRBase:MIPF0000567] were found to be differentially expressed in our analysis. osa-miR1861a, g, h, j and o are differentially expressed in both tissues, osa-miR1861b, d, f, i and l are only differentially expressed in leaf and osa-miR1861e, k and m are only differentially expressed in stem. The predicted targets of osa-MIR1861 include structural maintenance of chromosomes protein (*SMC5*), receptor-like protein kinase, glutathione peroxidase 4, mitotic control protein DIS3 and pentatricopeptide repeat –containing protein, which may indicate that osa-MIR1861 are regulating different important molecular functions in the rice plant. Functional information of non-conserved miRNA families is still not as readily available as conserved miRNA families. Therefore, analyses such as target prediction (Additional file 8) and significant GO pathway analysis (Additional files 9 and 10) will disclose more information of the former.

### Drought-responsive miRNAs with opposite expression patterns between tolerant and susceptible rice varieties

Interestingly, with our sequencing data, we found three families of conserved miRNAs that showed opposite differential expression patterns between drought-tolerant and drought-susceptible rice varieties. In leaf tissue, osa-miR397a/b [miRBase:MIPF0000120], osa-miR398b [miRBase:MIMAT0000983] and osa-miR408-5p [miRBase:MIMAT0022884] were down-regulated in Vandana and Aday Sel (drought-tolerant rice varieties) but were up-regulated in IR64 (drought-susceptible rice variety). However, the up-regulation of osa-miR397b, osa-miR398b and osa-miR408-5p in IR64 was >1.5 but <2.0 fold change. We also noticed that the expression level of osa-miR397a/b and osa-miR408-5p, under drought-free condition in leaf and stem tissues, was much higher in drought-tolerant rice varieties than the drought susceptible variety with the highest in Vandana.

It was previously reported that the expression of miR397 was down-regulated in drought-stressed rice leaf [19]. Furthermore, similar to our findings, miR397a/b were reported to be up-regulated in the sensitive genotype but down-regulated in the tolerant genotype of soybean during the water deficit condition [23]. miR397 is predicted to target β-fructofuranosidase, which takes part in starch and sucrose metabolism [19]. Due to the inverse relationship of miRNA and its target, the β-fructofuranosidase and its regulated biological process i.e. sucrose metabolism, would be up-regulated in the tolerant genotypes and down-regulated in the susceptible ones. It was hypothesized that the ability of a plant genotype to maintain a reasonable rate of the synthesis and metabolism of carbon-hydrogen compounds helps to protect itself against drought stress [52]. Therefore, this may be the important physiological factor that delimits the drought tolerant and susceptible phenotypes. miR397 is also predicted to target the laccase gene family which was reported to reduce root growth under dehydration in a knockout mutant [53]. Another study reported that overexpression of osa-miR397 enlarges grain size and promotes panicle branching, leading to an increase in overall yield of up to 25 % in field trial [54]. However, the specific role of miR397 in drought response and the reason for the opposite differential expression patterns observed between drought-tolerant and drought-susceptible rice varieties remains to be investigated.

miR398 was reported to be involved in antioxidant defence and respiratory electron transport of plant because it targets two closely related copper superoxide dismutases (*CSD1* and *CSD2*) and cytochrome C oxidase subunit V (*COX5b*) respectively [39, 16]. Drought stress enhances reactive oxygen species (ROS) in different cellular compartment, namely chroloplasts, peroxisomes, and mitochondria [55]. This may result in significant damage to plant cell structures. The increased formation of the superoxide anion O^2−^ in the response to stress is converted into less toxic molecules by superoxide dismutase proteins [49]. Hence, the down-regulation of osa-miR398b, observed in the tolerant rice varieties of our analysis, would lead to the increasing activities of CSDs to scavenge ROS. On the other hand, *COX5b*, another target of miR398, functions in the electron transport of the mitochondrial respiratory pathway [16]. The down-regulation of osa-miR398b, observed in the tolerant rice varieties of our analysis, would predictably lead to the increasing activities of COX5b and consequently the mitochondrial respiration under water deficit.

**Fig. 3:**
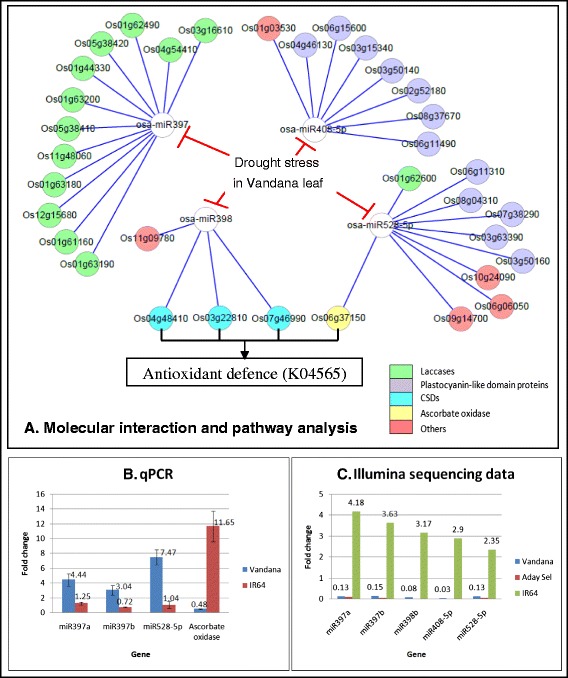
Molecular interaction, pathway and differential expression analyses of osa-miR397a/b, osa-miR398b, osa-miR408-5p and osa-miR528-5p between control and drought stress in the leaf tissue of different rice varieties. **a** This diagram shows, in the leaf of drought-tolerant rice varieties, drought suppresses the expression of these miRNAs, which in turn may collectively induce their previously validated copper-containing proteins. The opposite regulation happens in the leaf of drought-susceptible rice variety based on our Illumina sequencing data. **b** Normalized fold change (2^-∆∆Ct^) of four real time PCR validated genes with U6 snRNA as reference gene. Mean of triplicates of the normalized fold change of a qPCR validated gene is represented by a bar while the standard deviation of the triplicates is represented by an error bar. **c** Illumina sequencing data. Expression of control and drought stress was normalized on the basis of 1 M reads. Instead of |log_2_ fold change|, the normalized fold change is shown here for comparison with qPCR results

miR408 is known to targets plantacyanin. miR408-mediated regulation of plantacyanins is widely explored in response to copper and sucrose in *Arabidopsis* where the dynamics of miR408 contributes towards the maintenance of copper homeostasis [56–58]. Similar with miR397, miR408 was also previously found as down-regulated in drought-stressed rice leaf [19]. In chickpea, miR408 overexpressor lines showed increased drought tolerance presumably through the repression of plantacyanin transcript which in turn regulates Dehydration-Responsive Element Binding Protein 2A (*DREB2A*) and 1A (*DREB1A*) and other drought responsive genes [59]. Interestingly, by referring to degradome sequencing [28, 40, 60] and microarray data [61], these three families of miRNAs (MIR397, MIR398 and MIR408), together with MIR528, were validated to commonly target copper-containing proteins such as laccases, CSDs and plantacyanin in rice (Fig. 3a). Our sequencing results showed the opposite expression patterns of these three miRNA families to happen solely in leaf, the site of photosynthesis, between tolerant and susceptible rice varieties under drought stress. As copper is an essential micronutrient required for photosynthesis, it was proposed that, under conditions of low copper availability, higher plants prioritize the delivery of copper to plastocyanin and other essential copper proteins by down-regulation of nonessential or replaceable copper proteins (laccases, CSDs and plantacyanin) through the up-regulation of miR397, miR398 and miR408 [56]. Plastocyanin is one of the most abundant proteins in the thylakoid lumen and is essential for electron transfer between the cytochrome b_6_f complex and photosystem I [56]. In contrary to the collective up-regulation of miR397, miR398 and miR408 under low copper conditions, these genes together with miR528 were collectively down-regulated in the leaf of drought-tolerant rice varieties under drought treatment of our study. Since under copper deficit conditions, the co-regulation of these miRNAs is to maintain the optimal rate of photosynthesis [56], we postulate, based on analysis via Kyoto Encyclopedia of Genes and Genomes (KEGG), that the opposite co-regulation of these genes observed in the leaf of drought-tolerant varieties of our study may possibly function to activates plant’s survival-related processes such as antioxidant defence (K04565) (Fig. 3a). This findings indicate a link between copper homeostasis in leaf and drought response, which requires further study. However, other drought stress studies have also indicated the regulatory roles of these three miRNA families in other tissues of plants. For example, miR397, miR398 and miR408 were down-regulated only in root of peach [62] while miR398 and miR408 were strongly up-regulated in both shoot and root of *M. truncatula* [25].

We also found a non-conserved miRNA, osa-miR528-5p [miRBase:MIMAT0002884] that showed opposite differential expression pattern between tolerant and susceptible rice varieties. In leaf tissues, it was down-regulated in drought-tolerant rice varieties but up-regulated in drought-susceptible rice variety. However, the up-regulation of osa-miR528-5p is only 1.23 in log_2_ fold change. We also noticed that the expression level of osa-miR528-5p was significantly higher in the drought-tolerant rice varieties than the drought-susceptible rice variety under control condition. Besides targeting plastocyanin-like domain containing proteins, osa-miR528-5p, similar with osa-miR398b, is also involved in antioxidant defence by targeting ascorbate oxidase (Os06g37150) [28, 60, 61] (Fig. 3a). In maize, peroxidase (*POD*) is a predicted target of miR528. Wei et al. [63] reported that miR528 was down-regulated by drought in maize seedlings. RT-PCR analysis confirmed the inverse correlation of differential expression between miR528 and *POD* [63]. The up-regulation of *POD* would promote the removal of excessive H_2_O_2_ and alleviate the injury caused by ROS [52]. Due to the above function miR528-5p has been implicated in stress modulation both in biotic and abiotic stresses. It has been involved in both bacterial and fungal stress response where in a recent study involving *Bipolaris sorokiniana,* miR528-5p was highly induced in response to the fungal attack in both resistant and susceptible wheat cultivars [64] which contrast against the variety-specific expression observed under drought stress in our report.

We managed to amplify osa-miR397a/b, osa-miR528-5p with real-time PCR analysis. This qPCR analysis has otherwise shown that osa-miR397a/b and osa-miR528-5p are up-regulated in Vandana leaf but are not differentially expressed in IR64 leaf under drought stress. Figure 3b shows the fold change in expression levels of the genes in both rice varieties. The qPCR data fails to corroborate our Illumina sequencing data which showed that osa-miR397a/b, osa-miR398b, osa-miR408-5p and osa-miR528-5p were down-regulated in Vandana and Aday Sel leaf but up-regulated in IR64 leaf under drought stress (Fig. 3c).

The main factor that may contribute to this disparity of results is the different plant samples used for Illumina sequencing and qPCR analysis. This is partly due to the considerable time taken for the bioinformatic analysis of the small RNA sequencing data before these genes are selected for qPCR analysis. Therefore, fresh plant tissues were re-sampled with another round of drought stress treatment in the greenhouse. Although the drought treatments were identical, the well-known dynamic (fluctuation) expression of miRNAs when exposed to different durations of environmental stresses [65] is likely to be the factor that compromises the result’s consistency. For example, in contrary to our sequencing data, the expression levels of osa-miR408 transcript were reported to decrease significantly in sensitive rice cultivars, whereas they remain elevated in the tolerant rice cultivars at different time points of dehydration stress (1, 3 and 6 h) [58]. This is especially true as, in our greenhouse drought stress treatment, the time by which rice tissues are sampled is not specific enough as far as observation of leaf rolling is concerned.

Ascorbate oxidase (Os06g37150), one of the targets of osa-miR528 has also been amplified in our qPCR analysis. Our results show the negative correlation between these two genes in the Vandana leaf sample. However, in IR64 leaf sample, even when osa-miR528-5p is not differentially expressed, ascorbate oxidase is increasingly expressed under drought stress.

Even with the contrasting differential expression patterns observed among Illumina sequencing data, qPCR and previous report [58], for osa-miR397a/b, osa-miR398b, osa-miR408-5p and osa-miR528-5p, each result still points to the opposite, and perhaps functionally significant, expression profiles of these genes between the drought-tolerant and drought-susceptible rice varieties. More insights on the importance of these miRNAs in the regulation of rice drought response will possibly derived after further expression analyses (time course) is carried out on them in the rice varieties with different levels of drought tolerance.

### Enrichment of biological processes

**Fig. 4:**
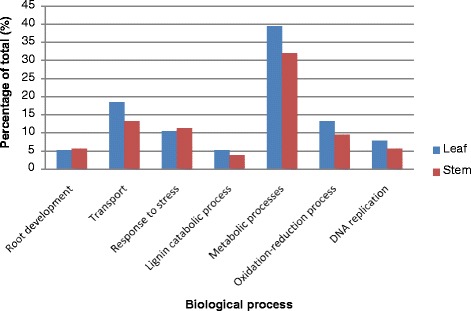
Bar chart showing the distribution of the enrichment of biological processes annotation between leaf and stem

A wide range of biological processes are found to be regulated by the target genes of the identified drought-responsive known miRNAs between leaf and stem, namely root development (5.3–5.7 %), cell transport (13.2–18.4 %), response to stress (10.5–11.3 %), lignin catabolic process (3.8–5.3 %), metabolic processes (32.1–39.5 %), oxidation-reduction process (9.4–13.2 %) and DNA replication (5.7–7.9 %). Zhou et al. [19] reported that the functions of the target genes of the drought stress-responsive miRNAs in rice cv. IRAT109 (*japonica*) include the morphological differentiation and development of shoot organs such as root, leaf and floral organs, hormone signal responses, miRNA regulation and abiotic stress responses. Differences in terms of rice subspecies database used for target prediction (therefore functional annotation) and the method used to analyze the enrichment of biological processes are likely to account for the discrepancy in some of the processes found to be enriched between both studies. Likewise, a similar distribution pattern was observed for the enrichment of biological processes between leaf and stem in our study (Fig. 4). Details of enrichment of biological processes for leaf and stem are shown respectively in Additional files 9 and 10, whereby drought-responsive miRNAs and their targets that were annotated with the biological process GO were documented. Differentially expressed miRNAs with target(s) that regulate several GO biological processes are shown in Table 2. For example, two drought-responsive miRNAs, osa-miR166e-3p [miRBase:MIMAT0000639] and osa-miR169r-3p* [miRBase:MIMAT0005967], are for the first time reported to predictably target alkaline neutral invertase and UDP-glucose 4-epimerase respectively, where each target are annotated to several GO biological processes including root development and carbohydrate metabolic process. In the perspective of GO biological process enrichment, these four drought-responsive miRNAs may function as major regulators in plant response towards drought stress, where osa-MIR397 [miRBase:MIPF0000120] is again highlighted (Table 2).

## Conclusions

Length distribution of small RNA reads and database mapping of clean reads show that our 12 small RNA libraries were highly enriched in miRNAs. High throughput sequencing is a very effective means to generate a digitalized expression profile of miRNAs due to its high specificity and sensitivity. In this aspect, expression levels of the conserved and non-conserved miRNA families were profiled where osa-miR397a/b, osa-miR408-5p and osa-miR528-5p were surprisingly found to be significantly more abundantly expressed in the leaf and stem tissues of drought-tolerant rice varieties under drought-free conditions. Twenty-six novel miRNA candidates involved in the regulation of diverse biological process were identified based on the detection of miRNA*. Out of their 110 predicted targets, we confirmed 16 targets from 5 novel microRNA candidates. In this study, we have identified 49 families of drought-responsive miRNAs in the 12 small RNA libraries generated from the leaf and stem tissues of three different rice varieties. Among them, 28 families are differentially expressed between both leaf and stem tissues while a few miRNA families (osa-MIR156, osa-MIR164 and osa-MIR827) are suggested to have tissue specificity in their differential expression. Notably, we also identify four miRNA families (osa-MIR397, osa-MIR398, osa-MIR408 and osa-MIR528) that display opposite differential expression patterns between the tolerant and susceptible rice varieties in the leaf tissues. These miRNA families have been known to be involved in regulating important biological processes such as starch metabolism, antioxidant defence, respiration and photosynthesis. With this information, it is very likely that these four miRNAs are playing a major regulatory role in drought response of rice, to an extent that the different tolerance levels of rice varieties towards drought are accounted. From the perspective of GO biological process enrichment, four drought-responsive miRNAs, osa-miR166e-3p, osa-miR166h-5p*, osa-miR169r-3p* and osa-miR397a/b, have target(s) that regulate several GO biological processes, indicating their potential function as major regulators in plant response towards drought stress. However, further experimentation is required to functionally study these miRNAs in order to unravel their delicate regulatory circuitry.

## Methods

### Plant materials and treatment

Three rice varieties with different drought tolerance: Vandana (drought-tolerant [66]; ancestry is 50 % tropical *japonica* and 50 % *aus*), Aday Sel (drought-tolerant [67]; *aus*) and IR64 (drought susceptible [67]; *indica*) were used. They were planted in potted soil in greenhouse subjected to natural solar radiation. All plants were grown under well-water conditions up to 3 weeks following emergence. For each rice variety, two experimental conditions were set up, which were drought and control. Drought treatment was given to 3 week old seedlings by stopping the watering according to the modified dry-down protocol in glasshouse [68]. The afternoon before drought treatment, all pots were fully watered (saturation). After draining overnight, the pots were enclosed in white plastic bags, around the stem to prevent direct soil-evaporation, and to avoid a rapid imposition of drought stress and homogenize the development of drought stress across replicated plants. A small tube was inserted in the plastic bags for re-watering pots. The pots were weighed after enclosing in plastic bags and this value was recorded as the initial target pot weight. Thereafter, the pots were weighed every morning at 8 AM and re-watering was only given to control plants to maintain their initial target pot weight. Drought treatment lasted until the leaves of the stressed plants started to roll (approximately 21 days). At that point, leaf and stem samples from each group were collected, snap frozen and kept in −80 °C until use. Taking account of the three experimental factors: the rice variety, experimental condition and plant tissue, a total of 12 small RNA libraries were constructed.

### Small RNA isolation, library construction and Solexa sequencing

Total RNA of the samples was extracted by using mirVana miRNA Isolation Kit (Invitrogen) according to the manufacturer’s protocol. Integrity of the total RNA samples was checked by Agilent 2100 Bioanalyzer (Agilent Technologies) and total RNA with RIN value of at least 6 (Additional file 3) was selected for library construction. Total RNA was separated by 15 % polyacrylamide gel electrophoresis (PAGE), and RNA molecules in the range of 18–30 nt were enriched and ligated with proprietary adapters to the 5′ and 3′ termini. The samples were reverse-transcribed and amplified by PCR to produce sequencing libraries. The small RNA library construction and Solexa sequencing was performed by Beijing Genomics Institute (BGI).

### Bioinformatics analysis

Overall workflow of BGI bioinformatics pipeline analysis for each of our small RNA libraries is shown in Additional file 1. Firstly in the pre-processing stage, Solexa 50 nt tags or raw reads with low sequencing quality, without 3′ adapter, without insert, with 5′ adapter, with length shorter than 18 nt after trimming the 3′ adapter sequence and with poly (A) tail were filtered out to produce clean reads. Next, the clean reads were perfectly mapped to BGI 9311 genome (ftp://ftp.genomics.org.cn/pub/ricedb/rice_update_data/genome/9311) [69] using the SOAP program [70]. To identify known miRNA sequences, clean reads were mapped to *Oryza sativa* miRNA sequences in miRBase version 21 (http://www.mirbase.org) [71]. The following two criteria have to be met to define a known miRNA and its expression; (i) sequences can be perfectly mapped onto the miRNA precursors in miRBase; (ii) sequences mapped to the mature miRNA in miRBase with at least 16 nt overlap allowing overhangs at the termini. Sequences were also mapped to rRNA, snRNA, snoRNA and tRNA from GenBank (http://www.ncbi.nlm.nih.gov/genbank) [72] and Sanger Rfam version 10.1 (ftp://ftp.sanger.ac.uk/pub/databases/Rfam) [73], repeat-associated sequences in Repbase RepeatMasker libraries (http://www.repeatmasker.org) [74], exon and intron of gene models (ftp://ftp.plantbiology.msu.edu/pub/data/Eukaryotic_Projects/o_sativa/annotation_dbs/pseudomolecules/version_7.0/all.dir/all.gff3) [75] from MSU Rice Genome Annotation Project. The remaining sequences that did not map to any databases were classified as unannotated reads. In addition, to ensure that every small RNA read only mapped to one annotation, the following priority order was set: rRNA, snRNA, snoRNA and tRNA (in which Genbank > Rfam) > known miRNA > repeat-associated > exon > intron.

To identify drought-responsive known miRNAs, the expression of miRNAs in the treated and control libraries were first normalized into TPM before the log_2_ fold change and the p-value were calculated for the miRNAs [76]. To minimize noise and improve accuracy, we only selected miRNAs with TPM ≥ 1 in at least one of the sample pair to be analyzed. The criteria to consider a miRNA to be drought-responsive are |log_2_ fold change| ≥ 2 and p-value ≤ 0.05 [76].

For novel miRNA prediction, the unannotated tags which can be mapped to reference genome, the tags that mapped to intron region and antisense exon region were used. Mireap (http://sourceforge.net/projects/mireap) software was used to predict novel miRNA by exploring the secondary structure, the Dicer cleavage site and the minimum free energy. Some key conditions are; (i) a candidate miRNA gene is defined if it can be folded into a secondary structure and mature miRNA is present in one arm or the hairpin precursor; (ii) the mature miRNA strand and its complementary strand (miRNA*) present with 2 nt 3′ overhang; (iii) hairpin precursor lacks large internal loops or bulges; (iv) the secondary structure of the hairpin is chemically stable, with the free energy of hybridization lower than or equal to −18 kcal/mol; and (v) the number of mature miRNA mapped to the predicted hairpin must be no fewer than 5. The expression of novel miRNA was defined by summing the count of most abundant tags with no more than 3 overhang mismatches at the termini and no mismatch in the middle of the alignment on the predicted precursor.

### Expression validation using quantitative real-time PCR

Three drought-responsive miRNAs and a target gene were selected for validation using qPCR as they were showing distinguished expression profiles among the leaf tissue of rice varieties with different drought tolerance. Firstly, total RNA of the samples was extracted by using mirVana miRNA Isolation Kit (Invitrogen) according to the manufacturer’s protocol and treated with RNase-free DNase 1 (Qiagen, Germany). Integrity of the total RNA samples was checked by Agilent 2100 Bioanalyzer (Agilent Technologies) and then 1 μg total RNA was reverse-transcribed using miScript II RT Kit (Qiagen, Germany) which allows the cDNA synthesis of miRNA, mRNA and other non-coding RNAs in the same reaction tube (20 μl). qPCR was performed on Bio-Rad iQ5 (USA) by using miScript SYBR Green PCR Kit (Qiagen, Germany). For each miRNA or non-coding RNA qPCR reaction, 2 μl cDNA template equivalent to 3 ng of total RNA was mixed with 10 μl of 2× QuantiTect SYBR Green PCR Master Mix (Qiagen, Germany), 2 μl of 10x miScript Universal Primer (Qiagen, Germany), 2 μl of forward primers (5pmol) and 4 μl if RNase-free water. For each mRNA qPCR reaction, the reaction mixture was almost similar except a higher concentration of cDNA (20 ng) and gene specific primer (5pmol) was used. PCR amplification protocol was as follows: 15 min at 95 °C for DNA Polymerase activation, 40 cycles consisting of 15 s at 94 °C, 30 s at 55 °C and 30 s at 70 °C. Melting curves were generated to verify the amplification specificity. Normalized fold change of a qPCR validated gene was calculated using Livak method (2^-∆∆Ct^) [77] with U6 snRNA as reference gene.

**Table:** 

Normalized Expression (∆∆Ct) of a target gene was calculated as follows: ∆Ct (drought) = Ct (target gene, drought) – Ct (U6 snRNA, drought) ∆Ct (control) = Ct (target gene, control) – Ct (U6 snRNA, control) Therefore, ∆∆Ct = ∆Ct (drought) - ∆Ct (control) (Sample loading differences are removed when relative quantity of a target gene is normalized by a relative quantity of the U6 snRNA reference gene.) Normalized fold change = 2 ^-∆∆Ct^	

Mean of triplicates of the normalized fold change of a qPCR validated gene is represented by a bar in Fig. 3b while the standard deviation of the triplicates is represented by an error bar. All the primers used are listed in Table 3.

**Table 3:** List of the primers used in quantitative real-time PCR analysis in the leaf tissue of Vandana and IR64 under control and drought conditions

Gene	Sequence
miR397a	Forward primer 5′ TCATTGAGTGCAGCGTTGATG 3′
miR397b	Forward primer 5′ TTATTGAGTGCAGCGTTGATG 3′
miR528-5p	Forward primer 5′ TGGAAGGGGCATGCAGAGGAG 3′
Ascorbate oxidase (Os06g37150)	Forward primer 5′ GGAGAGGACAGTTCGAGTGC 3′
	Reverse primer 5′ TAAGTCTTCCCCTGCTCGAC 3′
U6 snRNA	Forward primer 5′ TACAGATAAGATTAGCATGGCCCC-3′

### Target prediction and functional annotation

Target prediction for drought-responsive miRNAs and novel miRNA candidates against *indica* rice 9311 FGeneSH genes was conducted using psRNA Target: A Plant Small RNA Target Analysis Server [78] using default setting except the maximum expectation. This is the score of complementarity between miRNA and its target, which was adjusted in the range of 3.0–3.5 in our analysis. The functional annotation of the drought-responsive miRNAs to GO terms was performed using Blast2GO software version 2.6.5 [79]. GO Biological process and molecular function enrichments were analyzed using the combined graph function in Blast2GO. For molecular interaction and pathway analyses of selected miRNAs, previously validated target genes from degradome sequencing [28, 40, 60] and microarray data [61] were recorded and their molecular interactions were illustrated by Cytoscape 2.8.2 [80]. The target genes’ Rice Annotation Project (RAP) locus identifiers were used to search for their KEGG pathways [81].

### Validation of predicted targets of the 26 novel miRNA candidates

The predicted targets of the 26 novel miRNA candidates were further validated by searching the public degradome dataset from *Oryza sativa* ssp. Japonica [40] using CleaveLand software [41] with default parameters. The validated targets were identified based on similarity in locus identifier or target name between the target prediction and degradome analysis results.

## Availability of supporting data

Deep sequencing data from the 12 small RNA libraries have been deposited in the NCBI Sequence Read Archive (SRA) with accession numbers SRX1092587, SRX1092590, SRX1092594-SRX1092598, SRX1092626, SRX1092638, SRX1092681, SRX1092754 and SRX1092772 [http://www.ncbi.nlm.nih.gov/sra/?term=PRJNA289554].

## Additional files

Additional file 1:
**Flowchart for the analysis of sequence reads from small RNA libraries.** (PDF 189 kb) Additional file 2:
**Sequence pre-processing.** Tables show the statistics of filtering raw reads (Solexa 50 nt tags) generated from Solexa sequencing into clean reads for each small RNA library. (DOCX 21 kb) Additional file 3:
**Length distribution of small RNA reads.** Length distribution (length vs frequency percentage) of the small RNA reads from (A) Vandana, (B) Aday Sel and (C) IR64. (DOCX 140 kb) Additional file 4:
**Statistics of clean reads mapped to miRBase 21.** Table shows the statistics of clean reads mapped to the 592 precursors and 713 mature *Oryza sativa* miRNAs in miRBase 21. (DOCX 17 kb) Additional file 5:
**The Solexa sequencing expression profile of known** ***Oryza sativa*** **mature miRNAs deposited in miRBase 21.** Tables show mature miRNA names, mature miRNA sequences and the counts of expression level of (A) conserved and (B) non-conserved miRNA families from 12 small RNA libraries. (XLSX 68 kb) Additional file 6:
**Novel miRNA analysis.** (A) Novel miRNA candidates identified to be conserved in at least two libraries, (B) Novel miRNA candidates with miRNA*, (C) Predicted and validated targets (with GO annotation) of the 26 novel miRNA candidates. (XLSX 74 kb) Additional file 7:
**Secondary structures of novel miRNA candidates with miRNA*.** (DOCX 71 kb) Additional file 8:
**Differentially expressed known** ***Oryza sativa*** **miRNAs.** (A) Conserved miRNA families and (B) Non- conserved miRNA families that are differentially expressed under drought stress [82–89]. (DOCX 40 kb) Additional file 9:
**Enrichment of GO biological processes in leaf.** (DOCX 20 kb) Additional file 10:
**Enrichment of GO biological processes in stem.** (DOCX 23 kb)

## Abbreviations

QTL: Quantitative trait loci
miRNA: microRNA
nt: Nucleotide
tRNA: Transfer RNA
TPM: Transcripts per million
isomers: The population of variants of miRNAs coming from the same precursors
DCL1: DICER-LIKE 1
GO: Gene ontology
ARF: Auxin response factor
CSD: Copper superoxide dismutase
COX5b: Cytochrome C oxidase subunit V
ROS: Reactive oxygen species
POD: Peroxidase

## References

[1] Swamy BPM, Kumar A, Janeza TS (2011). Sustainable rice yield in water short drought prone environments: conventional and molecular approaches. Irrigation systems and practices in challenging environments.

[2] Wopereis MCS, Kropff MJ, Maligaya AR, Tuong TP (1996). Drought-stress responses of two lowland rice cultivars to soil water status. Field Crops Res.

[3] Chandra Babu R (2010). Breeding for drought resistance in rice: an integrated view from physiology to genomics. Electronic J Plant Breed.

[4] Chan CS, Zainudin H, Saad A, Azmi M (2012). Productive water use in aerobic rice cultivation. J Trop Agric Fd Sc.

[5] Schimid M, Davison TS, Henz SR, Pape UJ, Demar M, Vingron M, Scholkopf B, Weigel D, Lohmann JU (2005). A gene expression map of Arabidopsis thaliana development. Nat Genet.

[6] Rabbani MA, Maruyama K, Abe H, Khan MA, Katsura K, Ito Y, Yoshiwara K, Seki M, Shinozaki K, Yamaguchi-Shinozaki K (2003). Monitoring expression profiles of rice (Oryza sativa L.) genes under cold, drought and high-salinity stresses, and ABA application using both cDNA microarray and RNA gel blot analyses. Plant Physiol.

[7] Shinozaki K, Yamaguchi-Shinozaki K (2007). Gene networks involved in drought stress response and tolerance. J Exp Bot.

[8] Swamy BP, Vikram P, Dixit S, Ahmed HU, Kumar A (2011). Meta-analysis of grain yield QTL identified during agricultural drought in grasses showed consensus. BMC Genomics.

[9] Zhang JZ, Creelman RA, Zhu JK (2004). From laboratory to field. Using information from Arabidopsis to engineer salt, cold, and drought tolerance in crops. Plant Physiol.

[10] Bartels D, Sunkars R (2005). Drought and salt tolerance in plants. CRC Rev Plant Sci.

[11] Umezawa T, Fujita M, Fujita Y, Yamaguchi-Shinozaki K, Shinozaki K (2006). Engineering drought tolerance in plants: discovering and tailoring genes unlock the future. Curr Opin Biotechnol.

[12] Jeong DH, Park S, Zhai J, Gurazada SG, De Paoli E, Meyers BC, Green PJ (2011). Massive analysis of rice small RNAs: mechanistic implications of regulated microRNAs and variants for differential target RNA cleavage. Plant Cell.

[13] Jones-Rhoades MW, Bartel DP, Bartel B (2006). MicroRNAs and their regulatory roles in plants. Annu Rev Plant Biol.

[14] Sunkar R, Zhou X, Zheng Y, Zhang W, Zhu JK (2008). Identification of novel and candidate miRNAs in rice by high throughput sequencing. BMC Plant Biol.

[15] Liu HH, Tian X, Li YJ, Wu CA, Zheng CC (2008). Microarray-based analysis of stress-regulated microRNAs in Arabidopsis thaliana. RNA.

[16] Sunkar R, Zhu JK (2004). Novel and stress-regulated microRNAs and other small RNAs from Arabidopsis. Plant Cell.

[17] Li WX, Oono Y, Zhu J, He XJ, Wu JM, Iida K, Lu XY, Cui X, Jin H, Zhu JK (2008). The Arabidopsis NFYA5 transcription factor is regulated transcriptionally and posttranscriptionally to promote drought resistance. Plant Cell.

[18] Zhao B, Liang R, Ge L, Li W, Xiao H, Lin H, Ruan K, Jin Y (2007). Identification of drought-induced microRNAs in rice. Biochem Biophys Res Commun.

[19] Zhou L, Liu Y, Liu Z, Kong D, Duan M, Luo L (2010). Genome-wide identification and analysis of drought-responsive microRNAs in Oryza sativa. J Exp Bot.

[20] Xu C, Yang RF, Li WC, Fu FL (2010). Identification of 21 microRNAs in maize and their differential expression under drought stress. Afr J Biotechnol.

[21] Kantar M, Unver T, Budak H (2010). Regulation of barley miRNAs upon dehydration stress correlated with target gene expression. Funct Integr Genomics.

[22] Kantar M, Lucas SJ, Budak H (2011). miRNA expression patterns of Triticum dicoccoides in response to shock drought stress. Planta.

[23] Kulcheski FR, de Oliveira LF, Molina LG, Almerão MP, Rodrigues FA, Marcolino J, Barbosa JF, Stolf-Moreira R, Nepomuceno AL, Marcelino-Guimarães FC, Abdelnoor RV, Nascimento LC, Carazzolle MF, Pereira GA, Margis R (2011). Identification of novel soybean microRNAs involved in abiotic and biotic stresses. BMC Genomics.

[24] Lu S, Sun YH, Chiang VL (2008). Stress-responsive microRNAs in Populus. Plant J.

[25] Trindade I, Capitao C, Dalmay T, Fevereiro MP, Santos DM (2010). miR398 and miR408 are up-regulated in response to water deficit in Medicago truncatula. Planta.

[26] Arenas-Huertero C, Pérez B, Rabanal F, Blanco-Melo D, De la Rosa C, Estrada-Navarrete G, Sanchez F, Covarrubias AA, Reyes JL (2009). Conserved and novel miRNAs in the legume Phaseolus vulgaris in response to stress. Plant Mol Biol.

[27] Shen J, Xie K, Xiong L (2010). Global expression profiling of rice microRNAs by one-tube stem-loop reverse transcription quantitative PCR revealed important roles of microRNAs in abiotic stress responses. Mol Genet Genomics.

[28] Barrera-Figueroa BE, Gao L, Wu Z, Zhou X, Zhu J, Jin H, Liu R, Zhu JK (2012). High throughput sequencing reveals novel and abiotic stress-regulated microRNAs in the inflorescences of rice. BMC Plant Biol.

[29] Lee YS, Shibata Y, Malhotra A, Dutta A (2009). A novel class of small RNAs: tRNA-derived RNA fragments (tRFs). Genes Dev.

[30] Li T, Li H, Zhang YX, Liu JY (2011). Identification and analysis of seven H_2_O_2_-responsive miRNAs and 32 new miRNAs in the seedlings of rice (Oryza sativa L. ssp. indica). Nucleic Acids Res.

[31] Vaucheret H, Vazquez F, Crété P, Bartel DP (2004). The action of *ARGONAUTE1* in the miRNA pathway and its regulation by the miRNA pathway are crucial for plant development. Genes Dev.

[32] Mallory AC, Vaucheret H (2006). Functions of microRNAs and related small RNAs in plants. Nat Genet.

[33] Colaiacovo M, Bernardo L, Centomani I, Crosatti C, Giusti L, Orrù L, Tacconi G, Lamontanara A, Cattivelli L, Faccioli P (2012). A survey of microRNA length variants contributing to miRNome complexity in peach (Prunus persica L.). Front Plant Sci.

[34] Wu H, Neilson JR, Kumar P, Manocha M, Shankar P, Sharp PA, Manjunath N (2007). MiRNA profiling of naive, effector and memory CD8 T Cells. PLoS ONE.

[35] Ambros V, Bartel B, Bartel DP, Burge CB, Carrington JC, Chen X, Dreyfuss G, Eddy SR, Griffiths-Jones S, Marshall M, Matzke M, Ruvkun G, Tuschl T (2003). A uniform system for microRNA annotation. RNA.

[36] Lan Y, Su N, Shen Y, Zhang R, Wu F, Cheng Z, Wang J, Zhang X, Guo X, Lei C, Wang J, Jiang L, Mao L, Wan J (2012). Identification of novel miRNAs and miRNA expression profiling during grain development in indica rice. BMC Genomics.

[37] Meyers BC, Axtell MJ, Bartel B, Bartel DP, Baulcombe D, Bowman JL, Cao X, Carrington JC, Chen X, Green PJ, Griffiths-Jones S, Jacobsen SE, Mallory AC, Martienssen RA, Poethig RS, Qi Y, Vaucheret H, Voinnet O, Watanabe Y, Weigel D, Zhu JK (2008). Criteria for annotation of plant microRNAs. Plant Cell.

[38] Zhu QH, Spriggs A, Matthew L, Fan L, Kennedy G, Gubler F, Helliwell C (2008). A diverse set of microRNAs and microRNA-like small RNAs in developing rice grains. Genome Res.

[39] Jones-Rhoades MW, Bartel DP (2004). Computational identification of plant microRNAs and their targets, including a stress-induced miRNA. Mol Cell.

[40] Li YF, Zheng Y, Addo-Quaye C, Zhang L, Saini A, Jagadeeswaran G, Axtell MJ, Zhang W, Sunkar R (2010). Transcriptome-wide identification of microRNA targets in rice. Plant J.

[41] Addo-Quaye C, Miller W, Axtell MJ (2009). CleaveLand: a pipeline for using degradome data to find cleaved small RNA targets. Bioinformatics.

[42] Busse-Wicher M, Wicher KB, Kusche-Gullberg M (2014). The exostosin family: proteins with many functions. Matrix Biol.

[43] Xue T, Wang D, Zhang S, Ehlting J, Ni F, Jakab S, Zheng C, Zhong Y (2008). Genome-wide and expression analysis of protein phosphatase 2C in rice and Arabidopsis. BMC Genomics.

[44] Riechmann JL, Meyerowitz EM (1998). The AP2/EREBP family of plant transcription factors. Biol Chem.

[45] Herold A, Truant R, Wiegand H, Cullen BR (1998). Determination of the functional domain organization of the importin alpha nuclear import factor. J Cell Biol.

[46] Görlich D, Prehn S, Laskey RA, Hartmann E (1994). Isolation of a protein that is essential for the first step of nuclear protein import. Cell.

[47] Du P, Wu J, Zhang J, Zhao S, Zheng H, Gao G, Wei L, Li Y (2011). Viral infection induces expression of novel phased microRNAs from conserved cellular microRNA precursors. PLoS Pathog.

[48] Chen X, Zhang Z, Liu D, Zhang K, Li A, Mao L (2010). SQUAMOSA promoter-binding protein-like transcription factors: star players for plant growth and development. J Integr Plant Biol.

[49] De Lima JC, Loss-Morais G, Margis R (2012). MicroRNAs play critical roles during plant development and in response to abiotic stresses. Genet Mol Biol.

[50] Mallory AC, Bartel DP, Bartel B (2005). MicroRNA-directed regulation of Arabidopsis AUXIN RESPONSE FACTOR17 is essential for proper development and modulates expression of early auxin response genes. Plant Cell.

[51] Guilfoyle TJ, Hagen G (2001). Auxin response factors. J Plant Growth Regul.

[52] Ding Y, Tao Y, Zhu C (2013). Emerging roles of microRNAs in the mediation of drought stress response in plants. J Exp Bot.

[53] Cai X, Davis EJ, Ballif J, Liang M, Bushman E, Haroldsen V, Torabinejad J, Wu Y (2006). Mutant identification and characterization of the laccase gene family in Arabidopsis. J Exp Bot.

[54] Zhang YC, Yu Y, Wang CY, Li ZY, Liu Q, Xu J, Liao JY, Wang XJ, Qu LH, Chen F, Xin P, Yan C, Chu J, Li HQ, Chen YQ (2013). Overexpression of microRNA OsmiR397 improves rice yield by increasing grain size and promoting panicle branching. Nat Biotechnol.

[55] de Carvalho MH C (2008). Drought stress and reactive oxygen species: production, scavenging and signaling. Plant Signal Behav.

[56] Abdel-Ghany SE, Pilon M (2008). MicroRNA-mediated systemic down-regulation of copper protein expression in response to low copper availability in Arabidopsis. J Biol Chem.

[57] Ren L, Tang G (2012). Identification of sucrose-responsive microRNAs reveals sucrose-regulated copper accumulations in an SPL7-dependent and independent manner in Arabidopsis thaliana. Plant Sci.

[58] Mutum RD, Balyan SC, Kansal S, Agarwal P, Kumar S, Kumar M, Raghuvanshi S (2013). Evolution of variety-specific regulatory schema for expression of osa-miR408 in indica rice varieties under drought stress. FEBS J.

[59] Hajyzadeh M, Turktas M, Khawar KM, Unver T (2015). miR408 overexpression causes increased drought tolerance in chickpea. Gene.

[60] Wu L, Zhang Q, Zhou H, Ni F, Wu X, Qi Y (2009). Rice microRNA effector complexes and targets. Plant Cell.

[61] Xue LJ, Zhang JJ, Xue HW (2009). Characterization and expression profiles of miRNAs in rice seeds. Nucleic Acids Res.

[62] Eldem V, Çelikkol Akçay U, Ozhuner E, Bakır Y, Uranbey S, Unver T (2012). Genome-wide identification of miRNAs responsive to drought in peach (Prunus persica) by high-throughput deep sequencing. PLoS ONE.

[63] Wei L, Zhang D, Xiang F, Zhang Z (2009). Differentially expressed miRNAs potentially involved in the regulation of defense mechanism to drought stress in maize seedlings. Int J Plant Sci.

[64] Inal B, Türktaş M, Eren H, Ilhan E, Okay S, Atak M, Erayman M, Unver T (2014). Genome-wide fungal stress responsive miRNA expression in wheat. Planta.

[65] Jia X, Wang WX, Ren L, Chen QJ, Mendu V, Willcut B, Dinkins R, Tang X, Tang G (2009). Differential and dynamic regulation of miR398 in response to ABA and salt stress in Populus tremula and Arabidopsis thaliana. Plant Mol Biol.

[66] Venuprasad R, Lafitte HR, Atlin GN (2007). Response to direct selection for grain yield under drought stress in rice. Crop Sci.

[67] Swamy BP, Ahmed HU, Henry A, Mauleon R, Dixit S, Vikram P, Tilatto R, Verulkar SB, Perraju P, Mandal NP, Variar M, Robin S, Chandrababu R, Singh ON, Dwivedi JL, Das SP, Mishra KK, Yadaw RB, Aditya TL, Karmakar B, Satoh K, Moumeni A, Kikuchi S, Leung H, Kumar A (2013). Genetic, physiological, and gene expression analyses reveal that multiple QTL enhance yield of rice mega-variety IR64 under drought. PLoS ONE.

[68] Sinclair TR, Ludlow MM (1986). Influence of soil water supply on the plant water balance of four tropical grain legumes. Aust J Plant Physiol.

[69] Zhao W, Wang J, He X, Huang X, Jiao Y, Dai M, Wei S, Fu J, Chen Y, Ren X, Zhang Y, Ni P, Zhang J, Li S, Wang J, Wong GK, Zhao H, Yu J, Yang H, Wang J (2004). BGI-RIS: an integrated information resource and comparative analysis workbench for rice genomics. Nucleic Acids Res.

[70] Li R, Li Y, Kristiansen K, Wang J (2008). SOAP: short oligonucleotide alignment program. Bioinformatics.

[71] Griffiths-Jones S, Saini HK, van Dongen S, Enright AJ (2008). MiRBase: tools for microRNA genomics. Nucleic Acids Res.

[72] Benson DA, Cavanaugh M, Clark K, Karsch-Mizrachi I, Lipman DJ, Ostell J, Sayers EW (2013). GenBank. Nucleic Acids Res.

[73] Gardner PP, Daub J, Tate JG, Nawrocki EP, Kolbe DL, Lindgreen S, Wilkinson AC, Finn RD, Griffiths-Jones S, Eddy SR, Bateman A (2009). Rfam: updates to the RNA families database. Nucleic Acids Res.

[74] Jurka J, Kapitonov VV, Pavlicek A, Klonowski P, Kohany O, Walichiewicz J (2005). Repbase update, a database of eukaryotic repetitive elements. Cytogen Genome Res.

[75] Ouyang S, Zhu W, Hamilton J, Lin H, Campbell M, Childs K, Thibaud-Nissen F, Malek RL, Lee Y, Zheng L, Orvis J, Haas B, Wortman J, Buell CR (2007). The TIGR rice genome annotation resource: improvements and new features. Nucleic Acids Res.

[76] Audic S, Claverie JM (1997). The significance of digital gene expression profiles. Genome Res.

[77] Livak KJ, Schmittgen TD (2001). Analysis of relative gene expression data using real-time quantitative PCR and the 2(−Delta Delta C(T)) Method. Methods.

[78] Dai X, Zhao PX (2011). psRNATarget: a plant small RNA target analysis server. Nucleic Acids Res.

[79] Conesa A, Götz S, García-Gómez JM, Terol J, Talón M, Robles M (2005). Blast2GO: a universal tool for annotation, visualization and analysis in functional genomics research. Bioinformatics.

[80] Shannon P, Markiel A, Ozier O, Baliga NS, Wang JT, Ramage D, Amin N, Schwikowski B, Ideker T (2003). Cytoscape: a software environment for integrated models of biomolecular interaction networks. Genome Res.

[81] Kanehisa M, Goto S (2000). KEGG: kyoto encyclopedia of genes and genomes. Nucleic Acids Res.

[82] Reyes JL, Chua NH (2007). ABA induction of miR159 controls transcript levels of two MYB factors during Arabidopsis seed germination. Plant J.

[83] Liu Q, Zhang YC, Wang CY, Luo YC, Huang QJ, Chen SY, Zhou H, Qu LH, Chen YQ (2009). Expression analysis of phytohormone-regulated microRNAs in rice, implying their regulation roles in plant hormone signaling. FEBS Lett.

[84] Wu MF, Tian Q, Reed JW (2006). Arabidopsis microRNA167 controls patterns of ARF6 and ARF8 expression, and regulates both female and male reproduction. Development.

[85] Wang L, Mai YX, Zhang YC, Luo Q, Yang HQ (2010). MicroRNA171c-targeted SCL6-II, SCL6-III, and SCL6-IV genes regulate shoot branching in Arabidopsis. Mol Plant.

[86] Barrera-Figueroa BE, Gao L, Diop NN, Wu ZG, Ehlers JD, Roberts PA, Close TJ, Zhu JK, Liu R (2011). Identification and comparative analysis of drought-associated microRNAs in two cowpea genotypes. BMC Plant Biol.

[87] Meng Y, Ma X, Chen D, Wu P, Chen M (2010). MicroRNA-mediated signaling involved in plant root development. Biochem Biophys Res Commun.

[88] Bari R, Datt Pant B, Stitt M, Scheible WR (2006). PHO2, microRNA399, and PHR1 define a phosphate-signaling pathway in plants. Plant Physiol.

[89] Hackenberg M, Shi BJ, Gustafson P, Langridge P (2013). Characterization of phosphorus-regulated miR399 and miR827 and their isomirs in barley under phosphorus-sufficient and phosphorus-deficient conditions. BMC Plant Biol.

