# Supplementary figures and images for: Identification of four functionally important microRNA families with contrasting differential expression profiles between drought-tolerant and susceptible rice leaf at vegetative stage
Source: BMC Genomics. 2015 Sep 15;16(1):692. doi: 10.1186/s12864-015-1851-3 (PMC4570225; doi:10.1186/s12864-015-1851-3)

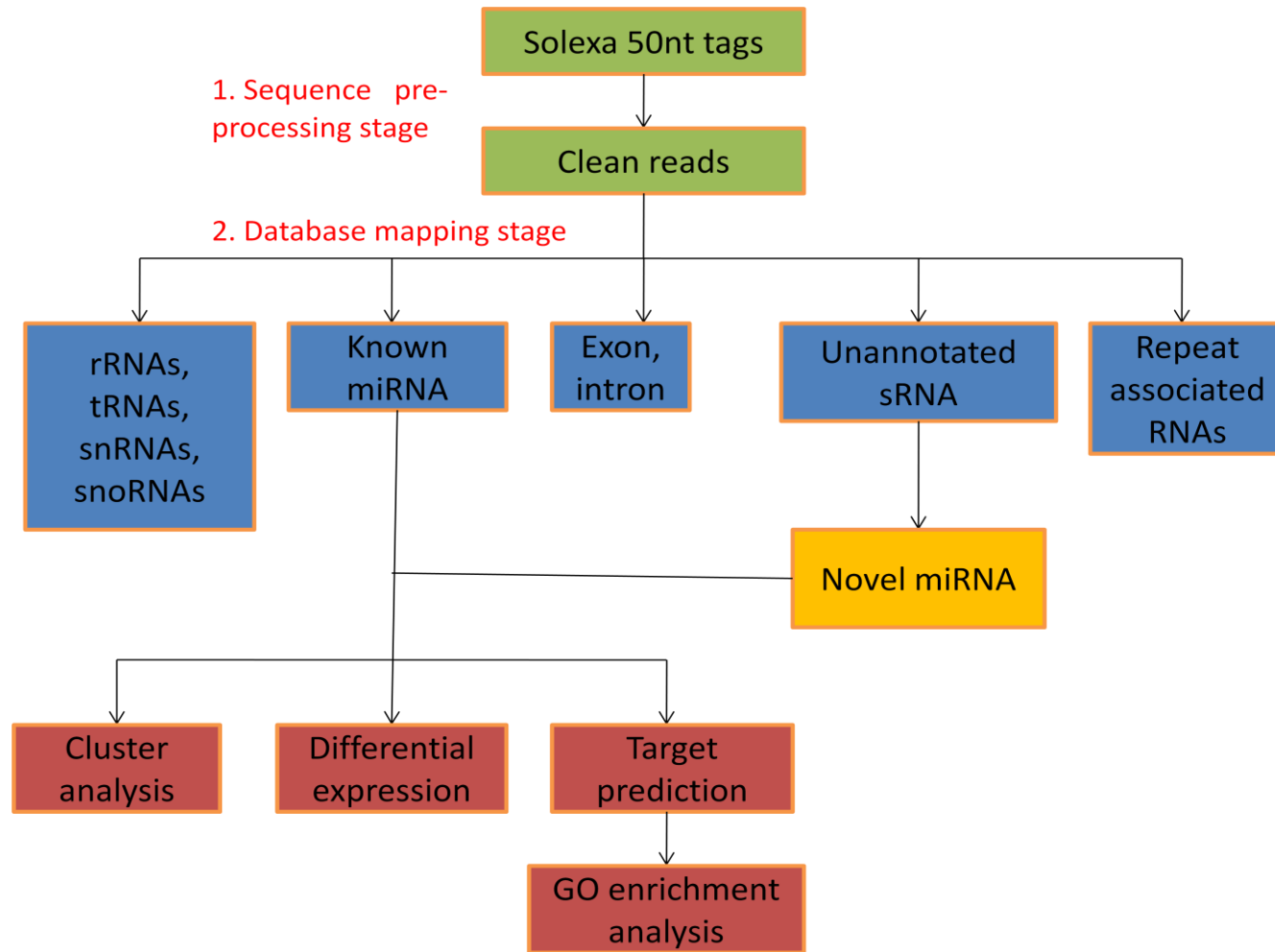

**Additional file 1. Flowchart for the analysis of sequence reads from small RNA libraries**

Supplement: Additional file 1: — Flowchart for the analysis of sequence reads from small RNA libraries. (PDF 189 kb) [file 12864_2015_1851_MOESM1_ESM.pdf]
