# Supplementary material for: Identification of four functionally important microRNA families with contrasting differential expression profiles between drought-tolerant and susceptible rice leaf at vegetative stage
Source: BMC Genomics. 2015 Sep 15;16(1):692. doi: 10.1186/s12864-015-1851-3 (PMC4570225; doi:10.1186/s12864-015-1851-3)
Supplement: Additional file 2: — Sequence pre-processing. Tables show the statistics of filtering raw reads (Solexa 50 nt tags) generated from Solexa sequencing into clean reads for each small RNA library. (DOCX 21 kb) [file 12864_2015_1851_MOESM2_ESM.docx]

| **Additional file 2. Sequence pre-processing** | **Vc leaf ^¥^** | | **Vc stem** | | **Vt leaf** | | **Vt stem** | |
| --- | --- | --- | --- | --- | --- | --- | --- | --- |
| **Type** | **Count** | **(%)** | **Count** | **(%)** | **Count** | **(%)** | **Count** | **(%)** |
| **Raw reads** | 19 143 613 |  | 16 621 511 |  | 16 899 609 |  | 19 422 425 |  |
| **High quality reads** | 19 107 233 | 100% | 16 589 083 | 100% | 16 867 060 | 100% | 19 378 785 | 100% |
| Reads without 3' adapter | 6 758 | 0.04% | 5 642 | 0.03% | 4 951 | 0.03% | 6 595 | 0.03% |
| Reads without insert | 3 025 | 0.02% | 3 161 | 0.02% | 5 110 | 0.03% | 3 176 | 0.02% |
| Reads with 5' adapter | 31 257 | 0.16% | 27 474 | 0.17% | 56 245 | 0.33% | 35 586 | 0.18% |
| Reads smaller than 18nt | 1 063 786 | 5.57% | 953 422 | 5.75% | 1 338 638 | 7.94% | 1 003 686 | 5.18% |
| Reads with Poly (A) tail | 1 506 | 0.01% | 825 | 0.00% | 1 244 | 0.01% | 1 147 | 0.01% |
| **Clean reads** | 18 000 901 | 94.21% | 15 598 559 | 94.03% | 15 460 872 | 91.66% | 18 328 595 | 94.58% |

|  | **Ac leaf ^¥^** | | **Ac stem** | | **At leaf** | | **At stem** | |
| --- | --- | --- | --- | --- | --- | --- | --- | --- |
| **Type** | **Count** | **(%)** | **Count** | **(%)** | **Count** | **(%)** | **Count** | **(%)** |
| **Raw reads** | 18 683 713 |  | 19 844 554 |  | 22 895 247 |  | 20 350 527 |  |
| **High quality reads** | 18 595 691 | 100% | 19 750 716 | 100% | 22 858 834 | 100% | 20 308 993 | 100% |
| Reads without 3' adapter | 4 277 | 0.02% | 4 559 | 0.02% | 10 761 | 0.05% | 7 738 | 0.04% |
| Reads without insert | 87 093 | 0.47% | 96 664 | 0.49% | 75 116 | 0.33% | 83 871 | 0.41% |
| Reads with 5' adapter | 33 936 | 0.18% | 44 857 | 0.23% | 58 023 | 0.25% | 68 819 | 0.34% |
| Reads smaller than 18nt | 3 788 750 | 20.37% | 5 463 044 | 27.66% | 5 666 855 | 24.79% | 4 404 170 | 21.69% |
| Reads with Poly (A) tail | 566 | 0.00% | 341 | 0.00% | 844 | 0.00% | 185 | 0.00% |
| **Clean reads** | 14 681 069 | 78.95% | 14 141 251 | 71.60% | 17 047 235 | 74.58% | 15 744 210 | 77.52% |

|  | **Rc leaf ^¥^** | | **Rc stem** | | **Rt leaf** | | **Rt stem** | |
| --- | --- | --- | --- | --- | --- | --- | --- | --- |
| **Type** | **Count** | **(%)** | **Count** | **(%)** | **Count** | **(%)** | **Count** | **(%)** |
| **Raw reads** | 18 222 011 |  | 18 238 171 |  | 17 952 889 |  | 20 640 460 |  |
| **High quality reads** | 18 171 491 | 100% | 18 186 077 | 100% | 17 904 966 | 100% | 20 585 832 | 100% |
| Reads without 3' adapter | 3 392 | 0.02% | 3 474 | 0.02% | 2 690 | 0.02% | 4 390 | 0.02% |
| Reads without insert | 1 000 | 0.01% | 644 | 0.00% | 2 071 | 0.01% | 738 | 0.00% |
| Reads with 5' adapter | 31 589 | 0.17% | 17 073 | 0.09% | 48 197 | 0.27% | 25 725 | 0.12% |
| Reads smaller than 18nt | 5 441 | 0.03% | 44 998 | 0.25% | 26 760 | 0.15% | 132 181 | 0.64% |
| Reads with Poly (A) tail | 2 061 | 0.01% | 1 310 | 0.01% | 3 329 | 0.02% | 656 | 0.00% |
| **Clean reads** | 18 128 008 | 99.76% | 18 118 578 | 99.63% | 17 821 919 | 99.54% | 20 422 142 | 99.20% |

Tables show the statistics of filtering raw reads (Solexa 50nt tags) generated from Solexa sequencing into clean reads for each small RNA library.

¥ V = Vandana, A = Aday Sel, R = IR64; c = control, t = drought treatment
