# Supplementary material for: Identification of four functionally important microRNA families with contrasting differential expression profiles between drought-tolerant and susceptible rice leaf at vegetative stage
Source: BMC Genomics. 2015 Sep 15;16(1):692. doi: 10.1186/s12864-015-1851-3 (PMC4570225; doi:10.1186/s12864-015-1851-3)
Supplement: Additional file 3: — Length distribution of small RNA reads. Length distribution (length vs frequency percentage) of the small RNA reads from (A) Vandana, (B) Aday Sel and (C) IR64. (DOCX 140 kb) [file 12864_2015_1851_MOESM3_ESM.docx]

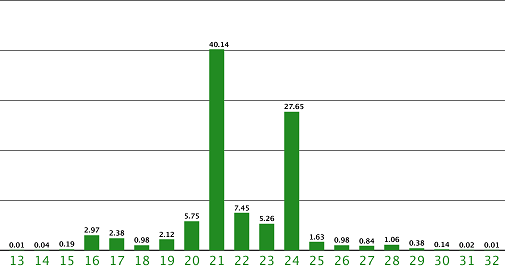

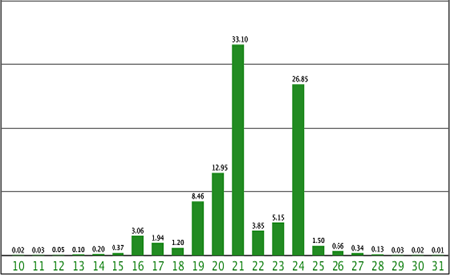


Drought, stem, RIN number=7.6

Drought, leaf, RIN number=7.0

Control, stem, RIN number=7.0

Control, leaf, RIN number=6.1

A. Length distribution (length vs frequency percentage) of the small RNA reads from Vandana


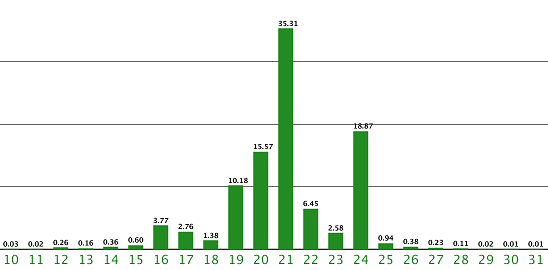

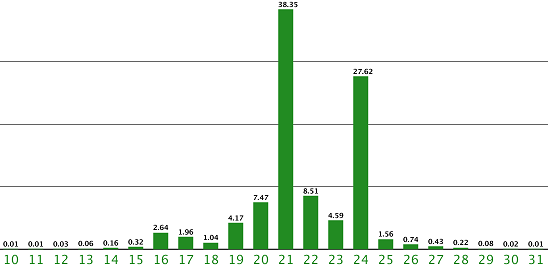


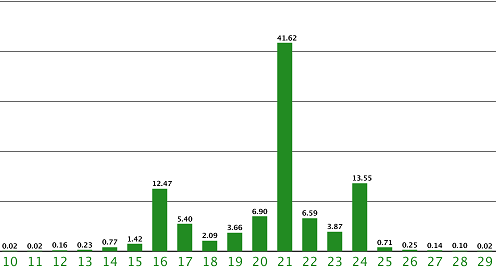

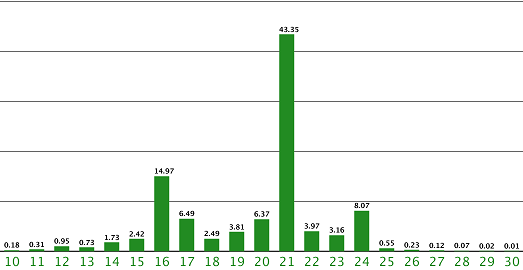


Drought, stem, RIN number=6.5

Control, stem, RIN number=7.7

Control, leaf, RIN number=6.0

B. Length distribution (length vs frequency percentage) of the small RNA reads from Aday Sel

Drought, leaf, RIN number=5.7


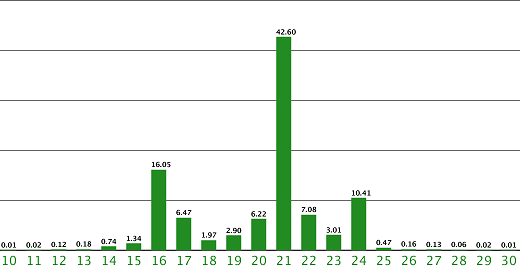

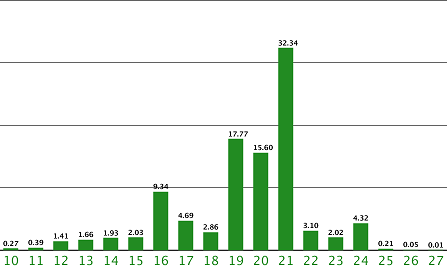


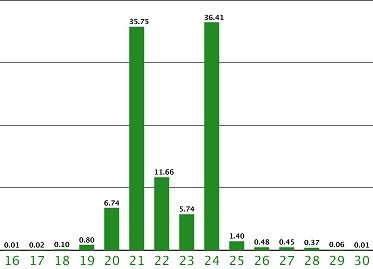

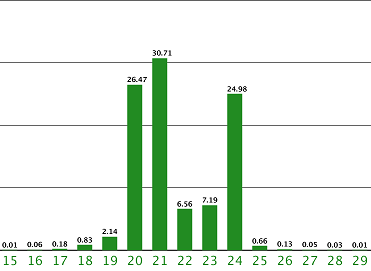


Drought, stem, RIN number=6.7

Drought, leaf, RIN number=6.0

Control, stem, RIN number=9.0

Control, leaf, RIN number=6.2

C. Length distribution (length vs frequency percentage) of the small RNA reads from IR64


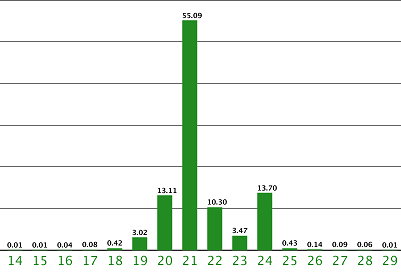

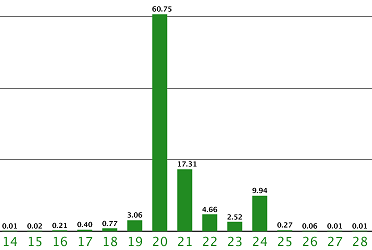


**Additional file 3. Length distribution of small RNA reads**
