# Supplementary material for: Identification of four functionally important microRNA families with contrasting differential expression profiles between drought-tolerant and susceptible rice leaf at vegetative stage
Source: BMC Genomics. 2015 Sep 15;16(1):692. doi: 10.1186/s12864-015-1851-3 (PMC4570225; doi:10.1186/s12864-015-1851-3)
Supplement: Additional file 4: — Statistics of clean reads mapped to miRBase 21. Table shows the statistics of clean reads mapped to the 592 precursors and 713 mature Oryza sativa miRNAs in miRBase 21. (DOCX 17 kb) [file 12864_2015_1851_MOESM4_ESM.docx]

| **Additional file 4. Statistics of clean reads mapped to miRBase 21** | **miRNA** | **miRNA -5p** | **miRNA -3p** | **miRNA precursors** | **Unique reads matched to miRNA precursors** | **Total reads matched to miRNA precursors** |
| --- | --- | --- | --- | --- | --- | --- |
| **Known miRNA in miRBase 21** | **498** | **108** | **107** | **592** | **-** | **-** |
| Vandana (control, leaf) | 309 | 78 | 83 | 385 | 6758 | 6523839 |
| Vandana (control, stem) | 317 | 76 | 80 | 391 | 6020 | 4684184 |
| Vandana (drought, leaf) | 307 | 81 | 77 | 384 | 5838 | 3372801 |
| Vandana (drought, stem) | 313 | 81 | 80 | 390 | 6927 | 3284270 |
| Aday Sel (control, leaf) | 320 | 78 | 82 | 397 | 6764 | 6390189 |
| Aday Sel (control, stem) | 303 | 71 | 78 | 375 | 5269 | 6983984 |
| Aday Sel (drought, leaf) | 308 | 77 | 82 | 387 | 7118 | 8222522 |
| Aday Sel (drought, stem) | 297 | 77 | 75 | 372 | 4954 | 5491314 |
| IR64 (control, leaf) | 300 | 81 | 81 | 378 | 6469 | 6317317 |
| IR64 (control, stem) | 313 | 79 | 80 | 390 | 6171 | 4245300 |
| IR64 (drought, leaf) | 302 | 80 | 80 | 378 | 6514 | 8536169 |
| IR64 (drought, stem) | 291 | 76 | 77 | 366 | 5220 | 2527590 |

Table shows the statistics of clean reads mapped to the 592 precursors and 713 mature *Oryza sativa* miRNAs in miRBase 21.
