# Supplementary material for: Identification of four functionally important microRNA families with contrasting differential expression profiles between drought-tolerant and susceptible rice leaf at vegetative stage
Source: BMC Genomics. 2015 Sep 15;16(1):692. doi: 10.1186/s12864-015-1851-3 (PMC4570225; doi:10.1186/s12864-015-1851-3)
Supplement: Additional file 7: — Secondary structures of novel miRNA candidates with miRNA*. (DOCX 71 kb) [file 12864_2015_1851_MOESM7_ESM.docx]

1a) cand_mir_76-5p

10 20 30 40 50 60

A GA-- .-GAGGGAGGG G G AT--| CA

GGG GGCGCGGCGATGTGGGGGAGGCGCGAC GT GCGC GCG TTTTCG \

CCC CCGCGCCGCTACACCCCCTCCGCGCTG TA CGTG CGC GGAGGC A

A GCGG \ --------- G G GCGG^ AT

140 130 120 80 70

Chr05:23489313:23489457:+ 145(nt) -95.80(kcal/mol)

AGGGGAGGCGCGGCGATGTGGGGGAGGCGCGACGAGGGAGGGGTGGCGCGGCGATTTTTCGCAATACGGAGGGGCGCGCGGTGCGATGGTGGTGCGGACTACGGAGGAGGGTCGCGCCTCCCCCACATCGCCGCGCCGGCGCCCA

.(((..(((((((((((((((((((((((((((.........((.((((.(((..((((((.....))))))....))).)))).)).(((((....)))))........)))))))))))))))))))))))))))....))). (-92.09)

------GGCGCGGCGATGTGGGGGAGG_5-----------------------------------------------------------------------------------CGCGCCTCCCCCACATCGCCGCG_1

------GGCGCGGCGATGTGGGGGAGGCG_1---------------------------------------------------------------------------------CGCGCCTCCCCCACATCGCCGCGC_5

------GGCGCGGCGATGTGGGGGAGGCGC_1----------------------------------------------------------------------------------CGCCTCCCCCACATCGCC_2

-------GCGCGGCGATGTGGGGGAGGC_73-----------------------------------------------------------------------------------CGCCTCCCCCACATCGCCG_1

-------GCGCGGCGATGTGGGGGAGGCG_2-----------------------------------------------------------------------------------CGCCTCCCCCACATCGCCGC_3

--------CGCGGCGATGTGGGGGAG_2--------------------------------------------------------------------------------------CGCCTCCCCCACATCGCCGCG_81

--------CGCGGCGATGTGGGGGAGG_4--------------------------------------------------------------------------------------GCCTCCCCCACATCGCCG_1

--------CGCGGCGATGTGGGGGAGGC_3-------------------------------------------------------------------------------------GCCTCCCCCACATCGCCGCG_1

--------CGCGGCGATGTGGGGGAGGCG_276----------------------------------------------------------------------------------GCCTCCCCCACATCGCCGCGC_22

--------CGCGGCGATGTGGGGGAGGCGC_2------------------------------------------------------------------------------------CCTCCCCCACATCGCCGC_1

---------GCGGCGATGTGGGGGAGG_1---------------------------------------------------------------------------------------CCTCCCCCACATCGCCGCGCC_4

---------GCGGCGATGTGGGGGAGGCG_10--------------------------------------------------------------------------------------TCCCCCACATCGCCGCGCCGG_6

---------GCGGCGATGTGGGGGAGGCGC_467

---------GCGGCGATGTGGGGGAGGCGCGA_1

---------GCGGCGATGTGGGGGAGGCGCGAC_9

----------CGGCGATGTGGGGGAGGC_12

----------CGGCGATGTGGGGGAGGCG_52

----------CGGCGATGTGGGGGAGGCGC_130

----------CGGCGATGTGGGGGAGGCGCG_475

----------CGGCGATGTGGGGGAGGCGCGA_5

----------CGGCGATGTGGGGGAGGCGCGAC_15

----------CGGCGATGTGGGGGAGGCGCGACG_2

-----------GGCGATGTGGGGGAGGCGCG_3

-------------CGATGTGGGGGAGGCGCG_1

1b) cand_mir_76-5p

10 20 30 40 50

C T CGCC .-CCTCCTCCGTAGTCC| ACCA

GG GGG GGCGCGGCGATGTGGGGGAGGCGCGAC GC \

CC CCC CCGCGCCGCTACACCCCCTCCGCGCTG CG C

- T CT-- \ ---------------^ CTAC

. 140 130 120

Chr05:23489311:23489460:- 150(nt) -86.10(kcal/mol)

CGGTGGGCGCCGGCGCGGCGATGTGGGGGAGGCGCGACCCTCCTCCGTAGTCCGCACCACCATCGCACCGCGCGCCCCTCCGTATTGCGAAAAATCGCCGCGCCACCCCTCCCTCGTCGCGCCTCCCCCACATCGCCGCGCCTCCCCTCC

.((.(((....(((((((((((((((((((((((((((...............((.........))...(((((............((((....)))))))))............)))))))))))))))))))))))))))..))).)) (-81.23)

----------CGGCGCGGCGATGTGGGGGAG_6------------------------------------------------------------------------------------CGCGCCTCCCCCACATCGCCGCG_1

----------CGGCGCGGCGATGTGGGGGAGG_2-----------------------------------------------------------------------------------CGCGCCTCCCCCACATCGCCGCGC_5

----------CGGCGCGGCGATGTGGGGGAGGCG_1-----------------------------------------------------------------------------------CGCCTCCCCCACATCGCC_2

-----------GGCGCGGCGATGTGGGGGAGG_5-------------------------------------------------------------------------------------CGCCTCCCCCACATCGCCG_1

-----------GGCGCGGCGATGTGGGGGAGGCG_1-----------------------------------------------------------------------------------CGCCTCCCCCACATCGCCGC_3

-----------GGCGCGGCGATGTGGGGGAGGCGC_1----------------------------------------------------------------------------------CGCCTCCCCCACATCGCCGCG_81

------------GCGCGGCGATGTGGGGGAGGC_73------------------------------------------------------------------------------------GCCTCCCCCACATCGCCG_1

------------GCGCGGCGATGTGGGGGAGGCG_2------------------------------------------------------------------------------------GCCTCCCCCACATCGCCGCG_1

-------------CGCGGCGATGTGGGGGAG_2---------------------------------------------------------------------------------------GCCTCCCCCACATCGCCGCGC_22

-------------CGCGGCGATGTGGGGGAGG_4---------------------------------------------------------------------------------------CCTCCCCCACATCGCCGC_1

-------------CGCGGCGATGTGGGGGAGGC_3--------------------------------------------------------------------------------------CCTCCCCCACATCGCCGCGCC_4

-------------CGCGGCGATGTGGGGGAGGCG_276

-------------CGCGGCGATGTGGGGGAGGCGC_2

--------------GCGGCGATGTGGGGGAGG_1

--------------GCGGCGATGTGGGGGAGGCG_10

--------------GCGGCGATGTGGGGGAGGCGC_467

--------------GCGGCGATGTGGGGGAGGCGCGA_1

--------------GCGGCGATGTGGGGGAGGCGCGAC_9

---------------CGGCGATGTGGGGGAGGC_12

---------------CGGCGATGTGGGGGAGGCG_52

---------------CGGCGATGTGGGGGAGGCGC_130

---------------CGGCGATGTGGGGGAGGCGCG_475

---------------CGGCGATGTGGGGGAGGCGCGA_5

---------------CGGCGATGTGGGGGAGGCGCGAC_15

----------------GGCGATGTGGGGGAGGCGCG_3

------------------CGATGTGGGGGAGGCGCG_1

2a) cand_mir_281-5p

10 20 30 40 50 60 70

CC CT - A GA-- .-GAGGGAGGG G G AT--| CA

GGA AG GG GGG GGCGCGGCGATGTGGGGGAGGCGCGAC GT GCGC GCG TTTTCG \

CCT TC CC CCC CCGCGCCGCTACACCCCCTCCGCGCTG TA CGTG CGC GGAGGC A

A- C- G A GCGG \ --------- G G GCGG^ AT

160 150 140 130 90 80

Chr05:23489302:23489467:+ 166(nt) -102.40(kcal/mol)

CCGGACTAGGGAGGGGAGGCGCGGCGATGTGGGGGAGGCGCGACGAGGGAGGGGTGGCGCGGCGATTTTTCGCAATACGGAGGGGCGCGCGGTGCGATGGTGGTGCGGACTACGGAGGAGGGTCGCGCCTCCCCCACATCGCCGCGCCGGCGCCCACCGCTCTCCA

..(((..((((.(((..(((((((((((((((((((((((((((.........((.((((.(((..((((((.....))))))....))).)))).)).(((((....)))))........)))))))))))))))))))))))))))....))).)).)).))). (-98.69)

-----------------GGCGCGGCGATGTGGGGGA_1-------------------------------------------------------------------------------------CGCGCCTCCCCCACATCGCCGCGC_1

-----------------GGCGCGGCGATGTGGGGGAG_1-------------------------------------------------------------------------------------GCGCCTCCCCCACATCGCCGC_1

-----------------GGCGCGGCGATGTGGGGGAGG_6-------------------------------------------------------------------------------------CGCCTCCCCCACATCGCCG_1

------------------GCGCGGCGATGTGGGGGAGG_1-------------------------------------------------------------------------------------CGCCTCCCCCACATCGCCGCG_53

------------------GCGCGGCGATGTGGGGGAGGC_86------------------------------------------------------------------------------------GCCTCCCCCACATCGCCGCG_2

-------------------CGCGGCGATGTGGGGGAGG_4--------------------------------------------------------------------------------------GCCTCCCCCACATCGCCGCGC_17

-------------------CGCGGCGATGTGGGGGAGGC_3--------------------------------------------------------------------------------------CCTCCCCCACATCGCCGCGCC_15

-------------------CGCGGCGATGTGGGGGAGGCG_178------------------------------------------------------------------------------------CTCCCCCACATCGCCGCG_1

--------------------GCGGCGATGTGGGGGAGGC_3----------------------------------------------------------------------------------------TCCCCCACATCGCCGCGC_1

--------------------GCGGCGATGTGGGGGAGGCG_12--------------------------------------------------------------------------------------TCCCCCACATCGCCGCGCC_1

--------------------GCGGCGATGTGGGGGAGGCGC_657------------------------------------------------------------------------------------TCCCCCACATCGCCGCGCCGG_6

--------------------GCGGCGATGTGGGGGAGGCGCG_1-------------------------------------------------------------------------------------TCCCCCACATCGCCGCGCCGGC_1

--------------------GCGGCGATGTGGGGGAGGCGCGA_1

--------------------GCGGCGATGTGGGGGAGGCGCGAC_3

---------------------CGGCGATGTGGGGGAGGC_15

---------------------CGGCGATGTGGGGGAGGCG_29

---------------------CGGCGATGTGGGGGAGGCGC_61

---------------------CGGCGATGTGGGGGAGGCGCG_195

---------------------CGGCGATGTGGGGGAGGCGCGA_2

---------------------CGGCGATGTGGGGGAGGCGCGAC_3

----------------------GGCGATGTGGGGGAGGCGCG_2

2b) cand_mir_281-5p

10 20 30 40 50 60

--- G- C T CGCC .-CCTCCTCCGTAGTCC| ACCA

GGA AG GG GGG GGCGCGGCGATGTGGGGGAGGCGCGAC GC \

CCT TC CC CCC CCGCGCCGCTACACCCCCTCCGCGCTG CG C

AGG GA - T CT-- \ ---------------^ CTAC

160 150 140 130 70

Chr05:23489301:23489466:- 166(nt) -92.50(kcal/mol)

GGAGAGCGGTGGGCGCCGGCGCGGCGATGTGGGGGAGGCGCGACCCTCCTCCGTAGTCCGCACCACCATCGCACCGCGCGCCCCTCCGTATTGCGAAAAATCGCCGCGCCACCCCTCCCTCGTCGCGCCTCCCCCACATCGCCGCGCCTCCCCTCCCTAGTCCGGA

(((.((.((.(((....(((((((((((((((((((((((((((...............((.........))...(((((............((((....)))))))))............)))))))))))))))))))))))))))..))).))))..)))... (-87.63)

---------------CCGGCGCGGCGATGTGGGGGAGGC_2----------------------------------------------------------------------------------CGCGCCTCCCCCACATCGCCGCGC_1

----------------CGGCGCGGCGATGTGGGGGA_1--------------------------------------------------------------------------------------GCGCCTCCCCCACATCGCCGC_1

----------------CGGCGCGGCGATGTGGGGGAG_4--------------------------------------------------------------------------------------CGCCTCCCCCACATCGCCG_1

-----------------GGCGCGGCGATGTGGGGGA_1---------------------------------------------------------------------------------------CGCCTCCCCCACATCGCCGCG_53

-----------------GGCGCGGCGATGTGGGGGAG_1---------------------------------------------------------------------------------------GCCTCCCCCACATCGCCGCG_2

-----------------GGCGCGGCGATGTGGGGGAGG_6--------------------------------------------------------------------------------------GCCTCCCCCACATCGCCGCGC_17

------------------GCGCGGCGATGTGGGGGAGG_1---------------------------------------------------------------------------------------CCTCCCCCACATCGCCGCGCC_15

------------------GCGCGGCGATGTGGGGGAGGC_86--------------------------------------------------------------------------------------CTCCCCCACATCGCCGCG_1

-------------------CGCGGCGATGTGGGGGAGG_4-----------------------------------------------------------------------------------------TCCCCCACATCGCCGCGC_1

-------------------CGCGGCGATGTGGGGGAGGC_3----------------------------------------------------------------------------------------TCCCCCACATCGCCGCGCC_1

-------------------CGCGGCGATGTGGGGGAGGCG_178

--------------------GCGGCGATGTGGGGGAGGC_3

--------------------GCGGCGATGTGGGGGAGGCG_12

--------------------GCGGCGATGTGGGGGAGGCGC_657

--------------------GCGGCGATGTGGGGGAGGCGCG_1

--------------------GCGGCGATGTGGGGGAGGCGCGA_1

--------------------GCGGCGATGTGGGGGAGGCGCGAC_3

---------------------CGGCGATGTGGGGGAGGC_15

---------------------CGGCGATGTGGGGGAGGCG_29

---------------------CGGCGATGTGGGGGAGGCGC_61

---------------------CGGCGATGTGGGGGAGGCGCG_195

---------------------CGGCGATGTGGGGGAGGCGCGA_2

---------------------CGGCGATGTGGGGGAGGCGCGAC_3

----------------------GGCGATGTGGGGGAGGCGCG_2

3) cand_mir_205-5p

10 20 30 40 50

A TG TGAGAA AAA - - T GT--| CCAAAATCCT

TT CAA GAATC AGGCT GCAG CAA TAC GGAG \

GG GTT CTTAG TCTGA CGTT GTT GTG CCTC C

A GT TGTGC- ACG G A T AGCT^ CTTTACTTGT

110 100 90 80 70 60

Chr11:12716259:12716370:+ 112(nt) -25.90(kcal/mol)

ATTTGCAATGAGAAGAATCAAAAGGCTGCAGCAATTACGTGGAGCCAAAATCCTCTGTTCATTTCCTCCTCGAGTGTTTGATTGCGAGTCTGCAGATTCCGTGTTTGTGGGA

.((..(((......(((((...((((((((((((.(((..((((.....................))))....))).))).)))).)))))...))))).....)))..)). (-25.90)

------AATGAGAAGAATCAAAAGGCTG_1----------------------------------------------------TGCGAGTCTGCAGATTCCGTG_5

-------ATGAGAAGAATCAAAAGGCTGCA_1

----------AGAAGAATCAAAAGGCTGCAGC_27

----------AGAAGAATCAAAAGGCTGCAGCA_2

4a) cand_mir_125-5p

10 20 30 40 50

TG--| A TATTT

GGTATTCTATAAAC CTTATAAATGATGCACACTGAACAATATTACTATA \

CCATAAGATATTTG GAATATTTACTACGTGTGACTTGTTATAATGATAT G

GGAA^ C TTACA

110 100 90 80 70 60

Chr11:16816208:16816324:+ 117(nt) -73.30(kcal/mol)

TGGGTATTCTATAAACACTTATAAATGATGCACACTGAACAATATTACTATATATTTGACATTTATAGTAATATTGTTCAGTGTGCATCATTTATAAGCGTTTATAGAATACCAAGG

..((((((((((((((.(((((((((((((((((((((((((((((((((((...........))))))))))))))))))))))))))))))))))).)))))))))))))).... (-73.30)

------------------TTATAAATGATGCACACTGAA_7---------------------------------TGTTCAGTGTGCATCATTTAT_1

-------------------TATAAATGATGCACACTGAAC_1----------------------------------TTCAGTGTGCATCATTTATAA_23

--------------------ATAAATGATGCACACTGAAC_2-----------------------------------TCAGTGTGCATCATTTATAA_2

--------------------ATAAATGATGCACACTGAACA_51---------------------------------TCAGTGTGCATCATTTATAAG_2

----------------------AAATGATGCACACTGAACAAT_2

4b) cand_mir_125-5p

10 20 30 40 50

TT--| A GT

GGTATTCTATAAACGCTTATAAATGATGCACACTGAACAATATTACTATA AT \

CCATAAGATATTTGTGAATATTTACTACGTGTGACTTGTTATAATGATAT TA C

GAAC^ A AA

110 100 90 80 70 60

Chr11:16816206:16816322:- 117(nt) -76.60(kcal/mol)

TTGGTATTCTATAAACGCTTATAAATGATGCACACTGAACAATATTACTATAAATGTCAAATATATAGTAATATTGTTCAGTGTGCATCATTTATAAGTGTTTATAGAATACCCAAG

..((((((((((((((((((((((((((((((((((((((((((((((((((.((.....)).)))))))))))))))))))))))))))))))))))))))))))))))))).... (-76.60)

------------------TTATAAATGATGCACACTGAA_7---------------------------------TGTTCAGTGTGCATCATTTAT_1

-------------------TATAAATGATGCACACTGAAC_1----------------------------------TTCAGTGTGCATCATTTATAA_23

--------------------ATAAATGATGCACACTGAAC_2-----------------------------------TCAGTGTGCATCATTTATAA_2

--------------------ATAAATGATGCACACTGAACA_51---------------------------------TCAGTGTGCATCATTTATAAG_2

----------------------AAATGATGCACACTGAACAAT_2

5) cand_mir_49-5p

10 20 30 40 50

--| CGC A CTACAC

ACTTTATGT CATTGACTAAGCATTTCTAGTTCATAACAC GCTAAATGGG \

TGAAATACA GTGACTGATTCGTAAAGATCAAGTATTGTG CGATTTACCT A

CT^ ACC C ATACAA

. 110 100 90 80 70

Chr03:27047046:27047166:+ 121(nt) -72.30(kcal/mol)

ACTTTATGTCGCCATTGACTAAGCATTTCTAGTTCATAACACAGCTAAATGGGCTACACAAACATATCCATTTAGCCGTGTTATGAACTAGAAATGCTTAGTCAGTGCCAACATAAAGTTC

(((((((((...((((((((((((((((((((((((((((((.((((((((((.............)))))))))).))))))))))))))))))))))))))))))...))))))))).. (-72.30)

-----------------ACTAAGCATTTCTAGTTCATAACA_1-----------------------------------TGTTATGAACTAGAAATGCTT_1

-------------------TAAGCATTTCTAGTTCATAAC_1--------------------------------------TTATGAACTAGAAATGCTTAG_13

-------------------TAAGCATTTCTAGTTCATAACA_2-------------------------------------TTATGAACTAGAAATGCTTAGT_3

--------------------AAGCATTTCTAGTTCATAACA_34---------------------------------------TGAACTAGAAATGCTTAGTCA_3

--------------------AAGCATTTCTAGTTCATAACAC_1

---------------------AGCATTTCTAGTTCATAACAC_3

6a) cand_mir_478-5p

10 20 30

- ATCCA- --| T

GGGT TGGGTTT TGTGGGTGTAGGGGGGAC T

CCTA GCCTAGG GCACCCACGTCCCCCCTG A

A GTCCCC TT^ A

70 60 50 40

Chr06:1139925:1140000:+ 76(nt) -49.50(kcal/mol)

GGGTATCCATGGGTTTTGTGGGTGTAGGGGGGACTTAAGTCCCCCCTGCACCCACGTTGGATCCGCCCCTGATCCA

((((.....(((((((((((((((((((((((((....))))))))))))))))))..)))))))......)))). (-49.50)

-------CATGGGTTTTGTGGGTGTAGGGGG_1--------CCCCCTGCACCCACGTTGGATCC_2

---------TGGGTTTTGTGGGTGTAGGGGGGA_1-------CCCCTGCACCCACGTTGGATCCGC_2

----------GGGTTTTGTGGGTGTAGGGGGGA_8----------CTGCACCCACGTTGGATCCGC_1

-----------GGTTTTGTGGGTGTAGGGGGGA_1

------------GTTTTGTGGGTGTAGGGGGGA_4

6b) cand_mir_478-5p

10 20 30

.-ATGG TT GAC

GT TGTGGGTGTAGGGGG T

CG GCACCCACGTCCCCC C

\ ---- TT GAG

50 40

Chr10:14435763:14435864:- 102(nt) -48.20(kcal/mol)

GGATATCCATGGGTTTTGTGGGTGTAGGGGGGACTCGAGCCCCCTGCACCCACGTTGCATCCGCACTGGCGGGACTGATTGTTGACGCCTGAGGATTATGCT

((....))....((..(((((((((((((((........)))))))))))))))..)).(((.((..((((.(((.....)))..)))))).)))....... (-48.50)

--------ATGGGTTTTGTGGGTGTAGGGGGG_1

---------TGGGTTTTGTGGGTGTAGGGGGGA_2

----------GGGTTTTGTGGGTGTAGGGGG_1

----------GGGTTTTGTGGGTGTAGGGGGG_1

----------GGGTTTTGTGGGTGTAGGGGGGA_4

----------GGGTTTTGTGGGTGTAGGGGGGAC_3

------------GTTTTGTGGGTGTAGGGGGGA_1

---------------TTGTGGGTGTAGGGGGGACT_1

7) cand_mir_874-5p

10 20 30 40 50

----- T GAGA C A C | A

CC CGGCG CTTCCTCTTCCCGTT GG GAATTGATGG CTCAG--CGAGGG \

GG GCCGT GAAGGAGAAGGGCAG CC CTTAGCTACC GAGTC GTTCCC T

GCGAC C A--- T A A \ ^ C

180 170 160 150 140 60

Chr02:26225904:26226087:- 184(nt) -93.00(kcal/mol)

CCTCGGCGGAGACTTCCTCTTCCCGTTCGGAGAATTGATGGCCTCAGCGAGGGATCCCCTTGAGCTCGGGAGACGCATTGGGGCGCGGGCTCCGGCGGCCTATCGTCGTCAGCGGAATCGGACACTTAATCCTGACTGAGACCATCGATTCACCTGACGGGAAGAGGAAGATGCCGCGGCAGCG

((.(((((....(((((((((((((((.((.((((((((((.(((((((((((...))))))...(((((((((((..((((.(((((...)).))).))))..).))))..((....)).........))))))))))).)))))))))).)).))))))))))))))).))))).))..... (-88.63)

-------------------TTCCCGTTCGGAGAATTGATGG_1

--------------------TCCCGTTCGGAGAATTGATGG_479

--------------------TCCCGTTCGGAGAATTGATGGCC_6

-----------------------------------------------------------------------------------------------------------------------------------------------ATCGATTCACCTGA CGGGAAG_10

8a) cand-mir_124-3p

10 20 30 40 50

ACT G-| A CTCA AAACAG G T

CTAGT ATTACTCGTAACCATTGTCCACAA TTAT AGA TG TAA A

GATTA TAATGAGCATTGGTAACAGGTGTT AATA TCT AC ATT A

AAC AA^ A ATGC ATA--- G A

110 100 90 80 70 60

Chr11:16269084:16269199:+ 116(nt) -44.60(kcal/mol)

ACTCTAGTGATTACTCGTAACCATTGTCCACAAATTATCTCAAGAAAACAGTGGTAATAAATTAGCAATATCTCGTAATAAATTGTGGACAATGGTTACGAGTAATAAATTAGCAA

...(((((.((((((((((((((((((((((((.((((....(((......((.(((....))).))...)))....)))).))))))))))))))))))))))))..)))))... (-44.60)

---------ATTACTCGTAACCATTGTCCA_1-----------------------------------------------------TGGACAATGGTTACGAGTAAT_34

----------TTACTCGTAACCATTGTCCAC_1

-----------TACTCGTAACCATTGTCCACA_1

8b) cand-mir_124-3p

10 20 30 40 50

T AATTT-| T A TATT TAATTTA

GCT ATTACTCGTAACCATTGTCCACAA TTATT CGAGA GC \

TGA TAATGAGCATTGGTAACAGGTGTT AATAG GTTCT TG T

T GATCAC^ T A TT-- TCACCAT

110 100 90 80 70 60

Chr11:16269083:16269198:- 116(nt) -48.80(kcal/mol)

TGCTAATTTATTACTCGTAACCATTGTCCACAATTTATTACGAGATATTGCTAATTTATTACCACTGTTTTCTTGAGATAATTTGTGGACAATGGTTACGAGTAATCACTAGAGTT

.(((.....((((((((((((((((((((((((.(((((.(((((....((...............))..))))).))))).))))))))))))))))))))))))......))). (-48.80)

----------TTACTCGTAACCATTGTCCAC_13---------------------------------------------AATTTGTGGACAATGGTTACG_2

-----------TACTCGTAACCATTGTCCACA_1---------------------------------------------------TGGACAATGGTTACGAGTAAT_99

-----------------TAACCATTGTCCACAATTTAT_2

9a) cand_mir_132-3p

10 20 30 40 50

-- G GCA CTC A GA - T A A- A-| AG

AT CCTC TTCCAT TTGGCT CTC GC GCTCCT CTTG GC TGAGC TG A

TA GGAG GGGGTA AACCGA GAG CG CGAGGA GAAC TG ACTCG GC A

TA G GGA AAT G AG A C G AG GG^ AA

120 110 100 90 80 70 60

Chr11:18346164:18346286:- 123(nt) -53.20(kcal/mol)

ATGCCTCGCATTCCATCTCTTGGCTACTCGAGCGCTCCTTCTTGAGCATGAGCATGAGAAAACGGGGCTCAGAGTGCAAGCAGGAGCAGCGAGAGGAGCCAATAAATGGGGAGGGAGGGATAT

((.((((...((((((...((((((.(((..((((((((.((((.((.(((((.((......))..)))))..)).)))).)))))).))..))).))))))...))))))...)))).)).. (-53.20)

--------------------TGGCTACTCGAGCGCTCCTTC_6-------------------------------------CAGGAGCAGCGAGAGGAGCCAA_1

---------------------------------------------------------------------------------AGGAGCAGCGAGAGGAGCCAA_4

---------------------------------------------------------------------------------AGGAGCAGCGAGAGGAGCCAAT_48

---------------------------------------------------------------------------------AGGAGCAGCGAGAGGAGCCAATAA_2

----------------------------------------------------------------------------------GGAGCAGCGAGAGGAGCCAAT_1

9b) cand_mir_132-3p

10 20 30 40 50

-- G GCA CTC A GA - T A A- A-| AG

AT CCTC TTCCAT TTGGCT CTC GC GCTCCT CTTG GC TGAGC TG C

TA GGAG GGGGTA AACCGA GAG CG CGAGGA GAAC CG ACTCG GC A

TA G GGA AAT G AG A C G AG GG^ AA

120 110 100 90 80 70 60

Chr11:18371156:18371278:- 123(nt) -55.30(kcal/mol)

ATGCCTCGCATTCCATCTCTTGGCTACTCGAGCGCTCCTTCTTGAGCATGAGCATGAGCAAACGGGGCTCAGAGCGCAAGCAGGAGCAGCGAGAGGAGCCAATAAATGGGGAGGGAGGGATAT

((.((((...((((((...((((((.(((..((((((((.((((.((.(((((.((......))..)))))..)).)))).)))))).))..))).))))))...))))))...)))).)).. (-55.30)

--------------------TGGCTACTCGAGCGCTCCTTC_6-------------------------------------CAGGAGCAGCGAGAGGAGCCAA_1

---------------------------------------------------------------------------------AGGAGCAGCGAGAGGAGCCAA_4

---------------------------------------------------------------------------------AGGAGCAGCGAGAGGAGCCAAT_48

---------------------------------------------------------------------------------AGGAGCAGCGAGAGGAGCCAATAA_2

----------------------------------------------------------------------------------GGAGCAGCGAGAGGAGCCAAT_1

10a) cand_mir_322-3p

10 20 30 40 50

TG--| A TATTT

GGTATTCTATAAAC CTTATAAATGATGCACACTGAACAATATTACTATA \

CCATAAGATATTTG GAATATTTACTACGTGTGACTTGTTATAATGATAT G

GGAA^ C TTACA

110 100 90 80 70 60

Chr11:16816208:16816324:+ 117(nt) -73.30(kcal/mol)

TGGGTATTCTATAAACACTTATAAATGATGCACACTGAACAATATTACTATATATTTGACATTTATAGTAATATTGTTCAGTGTGCATCATTTATAAGCGTTTATAGAATACCAAGG

..((((((((((((((.(((((((((((((((((((((((((((((((((((...........))))))))))))))))))))))))))))))))))).)))))))))))))).... (-73.30)

-------------------TATAAATGATGCACACTGAAC_1--------------------------TAATATTGTTCAGTGTGCATC_1

--------------------ATAAATGATGCACACTGAA_1--------------------------------TTGTTCAGTGTGCATCATTTA_2

--------------------ATAAATGATGCACACTGAACA_16------------------------------TGTTCAGTGTGCATCATTTAT_1

----------------------AAATGATGCACACTGAACAAT_1-------------------------------TTCAGTGTGCATCATTTATAA_24

10b) cand_mir_322-3p

10 20 30 40 50

TT--| A GT

GGTATTCTATAAACGCTTATAAATGATGCACACTGAACAATATTACTATA AT \

CCATAAGATATTTGTGAATATTTACTACGTGTGACTTGTTATAATGATAT TA C

GAAC^ A AA

110 100 90 80 70 60

Chr11:16816206:16816322:- 117(nt) -76.60(kcal/mol)

TTGGTATTCTATAAACGCTTATAAATGATGCACACTGAACAATATTACTATAAATGTCAAATATATAGTAATATTGTTCAGTGTGCATCATTTATAAGTGTTTATAGAATACCCAAG

..((((((((((((((((((((((((((((((((((((((((((((((((((.((.....)).)))))))))))))))))))))))))))))))))))))))))))))))))).... (-76.60)

-------------------TATAAATGATGCACACTGAAC_1--------------------------TAATATTGTTCAGTGTGCATC_1

--------------------ATAAATGATGCACACTGAA_1--------------------------------TTGTTCAGTGTGCATCATTTA_2

--------------------ATAAATGATGCACACTGAACA_16------------------------------TGTTCAGTGTGCATCATTTAT_1

----------------------AAATGATGCACACTGAACAAT_1-------------------------------TTCAGTGTGCATCATTTATAA_24

11) cand_mir_75-3p

10 20 30 40

C C CT A - .-CA| AG

TCCC TCTC CCTCTTCACC CCACCACCACC ACCAG GC \

AGGG AGGG GGGGGAGTGG GGTGGTGGTGG TGGTC CG A

- T AG - T \ --^ AG

190 180 170 160 50

Chr05:23352490:23352682:+ 193(nt) -100.20(kcal/mol)

CTCCCCTCTCCTCCTCTTCACCACCACCACCACCACCAGCAGCAGAGAGCACCATCTCCATCCAATAATCCCCATGCTTGCGCACCACTCCCGGCCACATCCCGCGCGAGGACGAGGAGGAGGAGGAGGAGGGTGTGCTTGATCCGCGCTCCCGCCTGGTTGGTGGTGGTGGGGTGAGGGGGGAGGGATGGGA

.((((.((((..((((((((((.((((((((((((((((..((.....))....(((((.(((.....(((((.(((((((((....................)))))))..)).)).))))))))))).(((.((((.......)))).)))..))))).)))))))))))))))))))))..)))).)))) (-97.65)

-------------CTCTTCACCACCACCACCACCA_1

--------------TCTTCACCACCACCACCACCA_1

------------------------------------------------------------------------------------------------------------------------------------------------------------------GTGGTGGTGGGGTGAGGG_1

-----GTGGTGGTGGGGTGAGGGG_3

-----GTGGTGGTGGGGTGAGGGGGG_13

12) cand_mir_524-5p

10 20 30 40 50 60 70 80

GTT A - C ---- TTG--- .-ATGTTGAAAAAAATTAT -| TTAT

TTTTAATAGATGAC CC GTTGACTTTTGG ATGA TGT GCC ATAAAA ATGTCAT T

AAAATTATCTACTG GG CAACTGAAAACC TATT ACA CGG TATTTT TACAGTG T

GAC - A A AATC TTTATA \ ----------------- G^ TTGT

180 170 160 150 140 100 90

Chr03:3379216:3379398:- 183(nt) -55.20(kcal/mol)

GTTTTTTAATAGATGACACCGTTGACTTTTGGCATGATGTTTGGCCATGTTGAAAAAAATTATATAAAAATGTCATTTATTTTGTTGTGACATGTTTTATCATTAAAGAAATCTTTAAGTATGACTTATAGGCATATTTACACTAATTATACCAAAAGTCAACAGGGTCATCTATTAAAACAG

...((((((((((((((.((((((((((((((.(((((((...(((.................(((((((((((((..........))))))).))))))..((((((....))))))............)))......)))....)))).)))))))))))).))))))))))))))))... (-50.29)

-------AATAGATGACACCGTTGACTTTTG_1

----------AGATGACACCGTTGACTTTTG_2

----------AGATGACACCGTTGACTTTTGG_15

----------AGATGACACCGTTGACTTTTGGCA_3

-------------TGACACCGTTGACTTTTGGCA_4

---------------ACACCGTTGACTTTTGGCATG_1

-------------------------------------------------------------------------------------------------------------------------------------------------------- AAA GTCAACAGGGTCATCTAT_1

13) cand_mir_448-5p

10 20 30 40

GT TA---| TT C-- C CTTCC AA

TTATCTG AATTT ATGGA GATGCGGG GT TG A

GGTAGAC TTAGG TACTT CTACGTCC CA AC A

CT TAGAA^ T- ATC C CAC-- AA

90 80 70 60 50

Chr07:22652776:22652867:+ 92(nt) -20.10(kcal/mol)

GTTTATCTGTAAATTTTTATGGACGATGCGGGCGTCTTCCTGAAAAAACACACACCCCTGCATCCTATTCATTGGATTAAGATCAGATGGTC

..(((((((..(((((..(((((.((((((((.((.....((......))...)).))))))))...))))).))))).....))))))).. (-20.10)

--------GTAAATTTTTATGGACGATGC_1-----------------------CCCTGCATCCTATTCATTGGATTA_1

--------GTAAATTTTTATGGACGATGCGGG_2-----------------------------CTATTCATTGGATTAAGATCAG_1

---------TAAATTTTTATGGACGATGCG_4--------------------------------TATTCATTGGATTAAGATCAGA_1

---------TAAATTTTTATGGACGATGCGG_14--------------------------------TTCATTGGATTAAGATCAGATGG_1

---------TAAATTTTTATGGACGATGCGGG_2----------------------------------CATTGGATTAAGATCAGATGG_1

----------AAATTTTTATGGACGATGCG_1-------------------------------------ATTGGATTAAGATCAGATGGT_20

----------AAATTTTTATGGACGATGCGG_115-----------------------------------TTGGATTAAGATCAGATGGTC_3

----------AAATTTTTATGGACGATGCGGG_854

----------AAATTTTTATGGACGATGCGGGC_3

----------AAATTTTTATGGACGATGCGGGCG_1

-----------AATTTTTATGGACGATGCGGG_147

-----------AATTTTTATGGACGATGCGGGC_6

------------ATTTTTATGGACGATGCGGG_1

------------ATTTTTATGGACGATGCGGGC_6

------------ATTTTTATGGACGATGCGGGCG_10

-------------TTTTTATGGACGATGCGGGCG_2

---------------TTTATGGACGATGCGGGCGTC_2

-------------------TGGACGATGCGGGCGTCTTCC_1

14) cand_mir_82-3p

10 20 30 40 50 60

ATTT| C C GA ATCCAG GGA C- A

ATTC CTCTTCA TG GCACACATGGAAAGAAT GGTT ACCC TTTTGC T

TAAG GAGAAGT AC CGTGTGTACCTTTTTTA CCGA TGGG GAAACG A

TATT^ A A GG CCAGA- AA- AT T

120 110 100 90 80 70

Chr05:24249386:24249512:- 127(nt) -55.70(kcal/mol)

ATTTATTCCCTCTTCACTGGAGCACACATGGAAAGAATATCCAGGGTTGGAACCCCTTTTGCATATGCAAAGTAGGGTAAAGCCAGACCATTTTTTCCATGTGTGCGGCAATGAAGAGAGAATTTAT

....((((.(((((((.((..(((((((((((((((((......((((...((((.((((((....))))))..))))..)))).....)))))))))))))))))..)).))))))).)))).... (-55.70)

--------CCTCTTCACTGGAGCACACATGGA_1-------------------------------------------------------------TCCATGTGTGCGGCAATGAAGAG_1

----------TCTTCACTGGAGCACACATGGA_5-------------------------------------------------------------TCCATGTGTGCGGCAATGAAGAGA_13

----------TCTTCACTGGAGCACACATGGAAA_3------------------------------------------------------------CCATGTGTGCGGCAATGAAGAGAG_8

-------------------------------------------------------------------------------------------------CATGTGTGCGGCAATGAAGAGA_2

-------------------------------------------------------------------------------------------------CATGTGTGCGGCAATGAAGAGAG_10

-------------------------------------------------------------------------------------------------CATGTGTGCGGCAATGAAGAGAGA_91

--------------------------------------------------------------------------------------------------ATGTGTGCGGCAATGAAGAG_1

--------------------------------------------------------------------------------------------------ATGTGTGCGGCAATGAAGAGA_3

--------------------------------------------------------------------------------------------------ATGTGTGCGGCAATGAAGAGAG_8

--------------------------------------------------------------------------------------------------ATGTGTGCGGCAATGAAGAGAGA_4

---------------------------------------------------------------------------------------------------TGTGTGCGGCAATGAAGAG_1

---------------------------------------------------------------------------------------------------TGTGTGCGGCAATGAAGAGAG_4

---------------------------------------------------------------------------------------------------TGTGTGCGGCAATGAAGAGAGAAT_6

-----------------------------------------------------------------------------------------------------TGTGCGGCAATGAAGAGAGAA_1

15) cand_mir_427-5p

10 20 30 40 50

TAA-- C CT A - .-CA| AG

TTCTCTCCC TCTC CCTCTTCACC CCACCACCACC ACCAG GC \

AGGGGAGGG AGGG GGGGGAGTGG GGTGGTGGTGG TGGTC CG A

CACGG T AG - T \ --^ AG

. 200 190 180 170

Chr05:23352483:23352692:+ 210(nt) -109.70(kcal/mol)

TAATTCTCTCCCCTCTCCTCCTCTTCACCACCACCACCACCACCAGCAGCAGAGAGCACCATCTCCATCCAATAATCCCCATGCTTGCGCACCACTCCCGGCCACATCCCGCGCGAGGACGAGGAGGAGGAGGAGGAGGGTGTGCTTGATCCGCGCTCCCGCCTGGTTGGTGGTGGTGGGGTGAGGGGGGAGGGATGGGAGGGGAGGCAC

...(((((((((.((((..((((((((((.((((((((((((((((..((.....))....(((((.(((.....(((((.(((((((((....................)))))))..)).)).))))))))))).(((.((((.......)))).)))..))))).)))))))))))))))))))))..)))).)))))))))..... (-107.15)

--------------------CTCTTCACCACCACCACCACCA_10

-------------------------------------------------------------------------------------------------------------------------------------------------------------------------GTGGTGGTGGGGTGAGGGGGG_9

16) cand_mir_395-5p

10 20 30 40 50 60 70 80 90

- A G C C-- T G G ATTTTA A T .-TTTAAAAAATAGAAAT| TACA

ATATTTGGCGTTTA GACAA AT TGGTCAAACT AAA TTTCGA TA CAAT TTCG TG TTAGT GGTAA \

TATAAACTGCAAAT CTGTT TA ACCAGTTTGA TTT AAGGCT GT GTTA AAGC AT AATCG CCATT T

T C A A AAA T G A ------ - - \ ----------------^ CAGA

220 210 200 190 180 170 160 100

Chr02:23825955:23826178:+ 224(nt) -57.80(kcal/mol)

ATATTTGGCGTTTAAGACAAGATCTGGTCAAACTCAAATTTTCGAGTAGCAATATTTTATTCGATGTTTAGTTTTAAAAAATAGAAATGGTAATACATAGACTTACCTTGAAGAGTACTATATAATATGATTAAGTTGTTGGATTTTATAAACTCATGCTAATACGAAATTGATGGTCGGAATTTTAAAAGTTTGACCAAATATTGTCCTAAACGTCAAATATT

((((((((((((((.(((((.((.((((((((((.(((.((((((.((.((((......((((.((.(((((................(((((.........))))).....((((..((((.(((.((((......)))).))).)))).))))..))))))))))))))).)).)))))).)))...)))))))))).)).))))).)))))))))))))). (-54.02)

----------TTTAAGACAAGATCTGGTCAA_10

-------------------------------------------------------------------------------------------------------------------------------------------------------------------------------------------------TGACCAAATATTGTCCTAAACGTC_1

17) cand_mir_695-3p

10 20 30 40 50 60 70

A| G C A C CT T CTG A

GTTTA ACAG TCCAGA CTCCACT CAGATTCAACT TGGAGCTAAATTTAGGAGT GGAG TACCAAAT G

TAAAT TGTC AGGTTT GAGGTGA GTCTGGGTTGA ATTTCGATTTAAATCCTCA CCTC ATGGTTTA G

C^ A A C A AG T AAA C

150 140 130 120 110 100 90 80

Chr04:33809182:33809335:+ 154(nt) -87.50(kcal/mol)

AGTTTAGACAGCTCCAGAACTCCACTCCAGATTCAACTCTTGGAGCTAAATTTAGGAGTTGGAGCTGTACCAAATAGGCATTTGGTAAAACTCCTACTCCTAAATTTAGCTTTAGAAGTTGGGTCTGAAGTGGAGCTTTGGAACTGTATAAATC

.(((((.((((.((((((.(((((((.(((((((((((..(((((((((((((((((((.((((...((((((((....))))))))...)))).)))))))))))))))))))..))))))))))).))))))).)))))).)))).))))). (-87.50)

---------AGCTCCAGAACTCCACTCCAGATT_3----------------------------------------------------------------------------------------TCTGAAGTGGAGCTTTGGAA_5

-------------CCAGAACTCCACTCCAGATTCAAC_2------------------------------------------------------------------------------------TCTGAAGTGGAGCTTTGGAAC_190

---------------AGAACTCCACTCCAGATTCAACTC_6----------------------------------------------------------------------------------TCTGAAGTGGAGCTTTGGAACT_2

---------------------------------------------------------------------------------------------------------------------------TCTGAAGTGGAGCTTTGGAACTG_4

---------------------------------------------------------------------------------------------------------------------------TCTGAAGTGGAGCTTTGGAACTGT_4

18) cand_mir_934-3p

10 20 30 40 50

--| TT AA T A A C AAGT

TAA AAGCA TCAAA TTCTGCATGTCATCAGGCAAG AA TTAATAAT CT \

ATT TTCGT AGTTT GAGACGTGTAGTAGTCTGTTC TT AATTATTA GA A

AT^ TT GG C A A A AAAA

110 100 90 80 70 60

Chr06:464855:464973:- 119(nt) -51.50(kcal/mol)

TAATTAAGCAAATCAAATTTCTGCATGTCATCAGGCAAGAAAATTAATAATCCTAAGTAAAAAAGAATTATTAAATTACTTGTCTGATGATGTGCAGAGCTTTGAGGTGCTTTTTTATA

(((..(((((..(((((.(((((((((((((((((((((.((.((((((((.((.........)).)))))))).)).))))))))))))))))))))).)))))..)))))..))).. (-51.50)

------------------TTCTGCATGTCATCAGGCAAG_7-------------------------------------CTTGTCTGATGATGTGCA_5

------------------TTCTGCATGTCATCAGGCAAGA_1------------------------------------CTTGTCTGATGATGTGCAG_2

--------------------CTGCATGTCATCAGGCAAG_1-------------------------------------CTTGTCTGATGATGTGCAGA_1

--------------------CTGCATGTCATCAGGCAAGAA_25----------------------------------CTTGTCTGATGATGTGCAGAG_57

--------------------CTGCATGTCATCAGGCAAGAAA_1----------------------------------CTTGTCTGATGATGTGCAGAGC_13

------------------------------------------------------------------------------CTTGTCTGATGATGTGCAGAGCT_2

-------------------------------------------------------------------------------TTGTCTGATGATGTGCAGAG_1

-------------------------------------------------------------------------------TTGTCTGATGATGTGCAGAGC_6

--------------------------------------------------------------------------------TGTCTGATGATGTGCAGA_5

--------------------------------------------------------------------------------TGTCTGATGATGTGCAGAG_3

--------------------------------------------------------------------------------TGTCTGATGATGTGCAGAGC_15

--------------------------------------------------------------------------------TGTCTGATGATGTGCAGAGCT_13

--------------------------------------------------------------------------------TGTCTGATGATGTGCAGAGCTT_1

---------------------------------------------------------------------------------GTCTGATGATGTGCAGAGCT_1

----------------------------------------------------------------------------------TCTGATGATGTGCAGAGCTT_2

19) cand_mir_463-3p

10 20 30 40 50 60 70 80 90 100

T C AC TAATA A T T ----- A | TTT

CCTATGCACA AGGCCCTCGCGTGTACACA GTGTACACCAACTAAAAA AC AAAAGA TCTA GAAAATTCATACAT TTCAAT GTATTACAT--GTAC C

GGATACGTGT TCCGGGAGTGCACATGTGT CACGTGTGGTTGATTTTT TG TTTTTT AGAT TTTTTAAGTATGTG AAGTTA CATAATGTG CGTG A

- T A- TATAG G - C CATGA A \ ^ CAT

350 340 330 320 310 300 290 280 270 260 110

Chr08:16242203:16242561:- 359(nt) -152.50(kcal/mol)

TCCTATGCACACAGGCCCTCGCGTGTACACAACGTGTACACCAACTAAAAATAATAACAAAAAGATTCTATGAAAATTCATACATTTCAATAGTATTACATGTACTTTCATACGTGCAAAGTCGCATCTTCAAATTCATTCTACATAGAGAATAACAAAAAAGATAAAATTCTGACAAAATTGCAATCTTGAAACTGTCGATTTTTTTTGTTACGGCTAAAATATAATGAATTTGAGGTTAAGATTTTAACCCTAGGTGTAATACAATTGAAAGTACGTGTATGAATTTTTCTAGATTTTTTGGTGATATTTTTTAGTTGGTGTGCACATGTGTACACGTGAGGGCCTTTGTGCATAGG

.((((((((((.(((((((((((((((((((..((((((((((((((((((.....((.((((((.((((.((((((((((((((((((((.(((((((((((((........)))).(((((....(((((((((((((.((..(((....(((((((((((((.......(((((......((....))....))))))))))))))))))...)))...)).)))))))))))))....))))).........))))))))).)))))).....)))))))))))))).)))))))))).)).....)))))))))))))))))).))))))))))))))))))).)))))))))) (-150.44)

----------ACAGGCCCTCGCGTGTACACAACG_5

----------------------------------------------------------------------------------------------------------------------------------------------------------------------------TGTGTACACGTGAGGGCCTT_4

---------------TGTGTACACGTGAGGGCCTTT_2

20a) cand_mir_388-5p

10 20 30 40 50

- TTT-- .-AAAAAAAATTTAAT| TTT

GTTTTT TAATTTTAATCGTCCGTTTTATT TAGTA \

CAAAGG GTTAAAATTAGCAGGCAGAATAA ATCGT T

T CACAT \ --------------^ TAT

150 140 130 60

Chr01:40538858:40539014:- 157(nt) -39.20(kcal/mol)

GTTTTTTTTTAATTTTAATCGTCCGTTTTATTAAAAAAAATTTAATTAGTATTTTTATTGCTATTAGCTTATAAAATATGAATAGTACTTTGTGCGTGACTTATATTTTTTTAAAAAAAATAAATAAGACGGACGATTAAAATTGTACACGGAAACT

((((((...(((((((((((((((((((((((..............(((((.......))))).....(((.(((((((((...((((...)))).....)))))))))..)))........))))))))))))))))))))))).....)))))). (-35.35)

-----TTTTTAATTTTAATCGTCCGTT_1---------------------------------------------------------------------------------------------------ACGGACGATTAAAATTGTACACGG_1

-------TTTAATTTTAATCGTCCGTTT_2

-------TTTAATTTTAATCGTCCGTTTT_1

----------AATTTTAATCGTCCGTTTT_1

----------AATTTTAATCGTCCGTTTTAT_4

----------AATTTTAATCGTCCGTTTTATTA_3

----------AATTTTAATCGTCCGTTTTATTAA_3

-------------TTTAATCGTCCGTTTTATTAAAAA_1

20b) cand_mir_388-5p

10 20 30 40 50 60 70

- CTC A C A TTTT .-ATTAAAA A CA-| CA

TTCTGTG AATTTT ATCGT CGTTTTATTT AAA TTTTATAA AACAT AGT AG T

AAGGCAC TTGAAA TAGCA GCAGAATAAA TTT AAAATATT TTGTA TTA TC A

A AAA C A C TAT- \ ------- C TCA^ AA

160 150 140 130 120 80

Chr04:9955424:9955590:- 167(nt) -32.20(kcal/mol)

TTCTGTGCTCAATTTTAATCGTCCGTTTTATTTAAAATTTTTTTTATAAATTAAAAAACATAAGTCAAGCATAAACTACTATTCATGTTCTATCATTTTATAACAATAAAAATACTAATTATAAAATATTTTCAAATAAGACGAACGATCAAAGTTAAACACGGAAA

(((((((...((((((.(((((.((((((((((.(((....((((((((.......(((((.(((..((......))...))).)))))......((((((....)))))).......))))))))...))).)))))))))).))))).))))))...))))))). (-28.28)

------GCTCAATTTTAATCGTCCGTT_1

--------TCAATTTTAATCGTCCGTT_1

--------TCAATTTTAATCGTCCGTTTT_1

--------TCAATTTTAATCGTCCGTTTTAT_2

----------AATTTTAATCGTCCGTTTTA_2

----------AATTTTAATCGTCCGTTTTAT_4

----------AATTTTAATCGTCCGTTTTATT_1

20c) cand_mir_388-5p

10 20 30 40 50 60

- C CC .-TATTTTTTTATAATTAAC| T

TTTCGTGTCTAATTTTAAT GT GTTTTATTT ATTATATAA \

AAGGTACAGATTGAAATTA TA TAGAATAAA TAGTATATT T

A C TA \ ------------------^ A

160 150 140 130 70

Chr05:29874205:29874366:+ 162(nt) -34.40(kcal/mol)

TTTCGTGTCTAATTTTAATCGTCCGTTTTATTTTATTTTTTTATAATTAACATTATATAATTATTATATGATAAAAACATAAATAATACTTTATGTGTGACTTATTTTTTTGGTTTTTTAAGTTTTTTAAATAAGATATATCATTAAAGTTAGACATGGAAA

(((((((((((((((((((.((..(((((((((..................(((((((((...)))))))))....(((((((......)))))))..((((((..............))))))....)))))))))..)).))))))))))))))))))). (-31.41)

---------TAATTTTAATCGTCCGTTT_1----------------------------------------------------------------GTGTGACTTATTTTTTTGGT_1---------------- AGATATATCATTAAAGTTAGACA_1

----------AATTTTAATCGTCCGTTTTA_2

----------AATTTTAATCGTCCGTTTTAT_4

----------AATTTTAATCGTCCGTTTTATT_1

----------------------------------------------------------------------------------------------

------------------------------------------------------------------------------------------------------------------------------------

21) cand_mir_405-3p

10 20 30 40

TTC| CT A AA TTAA TA

AGGTGA TGAA CT ACATGCCTTT AAAT \

TTCACT ACTT GG TGTATGGGGA TTTA A

GCT^ -- G GA TG-- TT

70 60 50

Chr03:7914010:7914086:+ 77(nt) -21.10(kcal/mol)

TTCAGGTGACTTGAAACTAAACATGCCTTTTTAAAAATTAATTATTTGTAGGGGTATGTAGGGGTTCATCACTTTCG

...((((((..((((.((..((((((((((....((((.....))))..))))))))))..)).))))))))))... (-21.10)

-----------------TAAACATGCCTTTTTAAAAAT_1---ATTTGTAGGGGTATGTAGGGGTTC_1

----------------------------------------------TGTAGGGGTATGTAGGGGTT_1

----------------------------------------------TGTAGGGGTATGTAGGGGTTC_6

22) cand_mir_327-5p

10 20 30 40

-| C ACT T TG ATTC A

GT CCAATAT GCAATCTAGGATA GA GGATTAT ATAT T

CA GGTTATA CGTTGGATCCTAT CT CCTGATG TATA C

G^ A CAT C -- CT-- C

90 80 70 60 50

Chr12:15066098:15066190:+ 93(nt) -35.70(kcal/mol)

GTCCCAATATACTGCAATCTAGGATATGATGGGATTATATTCATATATCCATATTCGTAGTCCTCCTATCCTAGGTTGCTACATATTGGAACG

((.(((((((...(((((((((((((.((..(((((((....((((....))))..))))))))).)))))))))))))...))))))).)). (-35.70)

--------ATACTGCAATCTAGGATATGATGG_1----------------------------CTCCTATCCTAGGTTGCTACATATT_1

----------ACTGCAATCTAGGATATGAT_1

----------ACTGCAATCTAGGATATGATG_2

----------ACTGCAATCTAGGATATGATGG_4

----------ACTGCAATCTAGGATATGATGGG_1

----------ACTGCAATCTAGGATATGATGGGA_2

--------------------------------------------------------------

23a) cand_mir_296-5p

10 20 30 40

AAC| C T A A TG

TACT CCTCTGTCCCAGAATAAGTTAATC AGT CT GGATG \

ATGA GGAGACAGGGTCTTATTCAATTAG TCA GA CCTAC T

AGT^ A T C - AT

90 80 70 60 50

Chr07:27200499:27200592:+ 94(nt) -57.30(kcal/mol)

AACTACTCCCTCTGTCCCAGAATAAGTTAATCTAGTACTAGGATGTGTTACATCCAGCACTTGATTAACTTATTCTGGGACAGAGGAAGTATGA

...((((.((((((((((((((((((((((((.(((.((.(((((.....))))))).))).)))))))))))))))))))))))).))))... (-57.30)

----------TCTGTCCCAGAATAAGTTAAT_10------------------------------TTAACTTATTCTGGGACAGAG_1

-----------------------------------------------------------------TAACTTATTCTGGGACAGAGG_1

23b) cand_mir_296-5p

10 20 30 40

TCA A -| TA

TACTTCCTCTGTCCCAGAATAAGTTAATC AGTGCT GGATG \

ATGAGGGAGACAGGGTCTTATTCAATTAG TCATGA CCTAC A

TTG A T^ AC

90 80 70 60 50

Chr07:27200499:27200592:- 94(nt) -66.20(kcal/mol)

TCATACTTCCTCTGTCCCAGAATAAGTTAATCAAGTGCTGGATGTAACACATCCTAGTACTAGATTAACTTATTCTGGGACAGAGGGAGTAGTT

...(((((((((((((((((((((((((((((.(((((((((((.....))))).)))))).)))))))))))))))))))))))))))))... (-66.20)

----------TCTGTCCCAGAATAAGTTAAT_10------------------------------TTAACTTATTCTGGGACAGAG_1

-----------------------------------------------------------------TAACTTATTCTGGGACAGAGG_1

------------------------------------------------------------------AACTTATTCTGGGACAGAGGG_3

-------------------------------------------------------------------ACTTATTCTGGGACAGAGGGA_1

24a) cand_mir_251-5p

10 20 30 40 50

- A CAG .-TTTTGCCCCC| G

TGTGTT GTTTGGACGTTTTCC CGCGCTACAGTG GC C

ACACAA CAAACCTGCAAAAGG GCGCGATGTCAT CG A

G G ATA \ ----------^ C

230 220 210 200

Chr02:11325468:11325704:- 237(nt) -73.80(kcal/mol)

TGTGTTAGTTTGGACGTTTTCCCAGCGCGCTACAGTGTTTTGCCCCCGCGCACGCTTTTCAAACTACTACACGGTGTACTTTTTTGCAAAAAGTTTCTATACAAAAGTTGCTTAAAAAATCAAATTAATCCATTTTTGAAAAAAAAATTAGCTAATACTTAATTAATCACGTGTTAATGAACCGTTCCGTTTTCCGTGTTACTGTAGCGCGATAGGAAAACGTCCAAACGAACACAG

((((((.(((((((((((((((...((((((((((((..........((....)).............((((((...((......((...((((..((.......))..))))...................(((((....)))))....))................(((.(((....))))))...))...))))))))))))))))))...))))))))))))))).)))))). (-61.24)

----------TGGACGTTTTCCCAGCGCGCT_11

24b) cand_mir_251-5p

10 20 30 40 50

- G .-TTTTGCCCCC| G

TGTGTTCGTTTGGACGTTTTCCCA CGCGCTACAGTG GC C

ACACAAGCAAACCTGCAAAAGGGT GCGCGATGTCAT CG A

G A \ ----------^ C

230 220 210 200

Chr04:29021850:29022085:- 236(nt) -83.90(kcal/mol)

TGTGTTCGTTTGGACGTTTTCCCAGCGCGCTACAGTGTTTTGCCCCCGCGCACGCTTTTCAAACTACTACACGGTGTACTTTTTTGCAAAAGTTTTTATACAAAGTTGTTTAAAAAAATCAAATTAATCCATTTTTGAAAAAAAAATTAGCTAATACTTAATTAATCACGTATTAATAAACCGTTCCGTTTTCCGTGTTACTGTAGCGCGATGGGAAAACGTCCAAACGAACACAG

((((((((((((((((((((((((.((((((((((((..........((....)).............((((((...((.(((((.((((((((((((.(((....))).)))))...............))))))).)))))...(((..((((((............)))))).)))........))...)))))))))))))))))).)))))))))))))))))))))))). (-77.26)

----------TGGACGTTTTCCCAGCGCGCT_11

24c) cand_mir_251-5p

10 20 30 40 50

- C .-TTTTACCCCC| G

TGTGTTCGTTTGGACGTTTTCCCAGCGCGCTA AGTG GC C

ACACAAGCAAACCTGCAAAAGGGTCGCGCGAT TCAT CG A

G A \ ----------^ C

230 220 210 200

Chr07:3653968:3654204:- 237(nt) -85.50(kcal/mol)

TGTGTTCGTTTGGACGTTTTCCCAGCGCGCTACAGTGTTTTACCCCCGCGCACGCTTTTCAAACTACTACACGGTGTACTTTTTTGCAAAAAGTTTCTATACAAAAGTTGCTTAAAAAATCAAATTAATTCATTTTTGAAAAAAAAATTAGCTAATACTTAATTAATCACGTGTTAATGAACCGTTCCGTTTTCCGTGTTACTATAGCGCGCTGGGAAAACGTCCAAACGAACACAG

((((((((((((((((((((((((((((((((.((((..........((....)).............((((((...((......((...((((..((.......))..))))...................(((((....)))))....))................(((.(((....))))))...))...)))))))))).)))))))))))))))))))))))))))))))). (-72.94)

----------TGGACGTTTTCCCAGCGCGCT_11-------------TAGCGCGCTGGGAAAACGTCC_1

24d) cand_mir_251-5p

10 20 30 40 50

- C .-AA| GAAAACG C

TG GTTCGTTTGGACGTTTTCCCAGCGCGCTACAGT CACG GAA \

AC CAAGCAAACCTGCAAAAGGGTCGCGCGATGTCA GTGC CTT G

G A \ --^ ACAATTA G

. 230 220 210 60

Chr01:27179099:27179338:+ 240(nt) -92.30(kcal/mol)

TGCGTTCGTTTGGACGTTTTCCCAGCGCGCTACAGTAACACGGAAAACGGAACGGTTCATTAACACGTGATTAATTAAGTATTAGCTAATTTTTTTTTCAAAAATGGATTAATTTGATTTTTTTTTAAGCAACTTTTGTATAAAAACTTTTTGCAAAAAAGTACACCGTGTAGTAGTTTGAAAAGCGTGCGCGGGGGCAAAACACTGTAGCGCGCTGGGAAAACGTCCAAACGAACACAG

((.(((((((((((((((((((((((((((((((((..((((.......(((...))).......))))...............(((...(((((((.((((((..((((.....))))..((((((.((((...)))).)))))).)))))).)))))))....(((((((((..........)).))))))).))).....))))))))))))))))))))))))))))))))).)). (-85.45)

----------TGGACGTTTTCCCAGCGCGCT_5

24e) cand_mir_251-5p

10 20 30 40 50

- C .-AA| GAAAACG C

TGTG TCGTTTGGACGTTTTCCCAGCGCGCTACAGT CACG GAA \

ACAC AGCAAACCTGCAAAAGGGTCGCGCGATGTCA GTGC CTT G

G A \ --^ ACAATTA G

230 220 210 60

Chr03:2947213:2947449:- 237(nt) -88.80(kcal/mol)

TGTGCTCGTTTGGACGTTTTCCCAGCGCGCTACAGTAACACGGAAAACGGAACGGTTCATTAACACGTGATTAATTAAGTATTAGCTAATTTTTTTTTTAAAAATGGATTAATTTGATTTTTTTAAGTAACTTTTGTATAGAAACTTTTTGCAAAAAAATACACCCTGTAGTAGTTTGAAAAACGTGCGCGGGACAAAACACTGTAGCGCGCTGGGAAAACGTCCAAACGAACACAG

((((.(((((((((((((((((((((((((((((((..((((.......(((...))).......)))).........(((((...........(((..((((((.((.....)).))))))..)))....(((((((...........))))))).)))))..(((((.(((((((...)))).)))))))).......))))))))))))))))))))))))))))))).)))). (-82.10)

----------TGGACGTTTTCCCAGCGCGCT_5

25) cand_mir_72-3p

10 20 30 40

ATA| G A CTC GC

TTTTCTAGAGACGATT ACTTAAGATGGC GC TGAAGAT A

AAAAGGTCTCTGCTAG TGAATTCTACCG CG ACTTTTG C

ATA^ - G AAT TA

90 80 70 60 50

Chr04:21833041:21833135:- 95(nt) -50.50(kcal/mol)

ATATTTTCTAGAGACGATTGACTTAAGATGGCAGCCTCTGAAGATGCACATGTTTTCATAAGCGGCCATCTTAAGTGATCGTCTCTGGAAAAATA

...((((((((((((((((.((((((((((((.((...(((((((......)))))))...)).))))))))))))))))))))))))))))... (-50.50)

---------AGAGACGATTGACTTAAGATGG_1-------------------------------GCCATCTTAAGTGATCGTCTC_5

---------AGAGACGATTGACTTAAGATGGCA_1--------------------------------ATCTTAAGTGATCGTCTCT-2

----------GAGACGATTGACTTAAGATGGCA_1

-----------AGACGATTGACTTAAGATGGCA_1

------------GACGATTGACTTAAGATGGCAG_1

-------------------------AGATGGCAGCCTCTGAAGATGCAC_1

26) cand_mir_407-3p

10 20 30 40 50

TC-| C A CTACAC

GC ATTGACTAAGCATTTCTAGTTCATAACAC GCTAAATGGG \

CG TGACTGATTCGTAAAGATCAAGTATTGTG CGATTTACCT A

AAC^ - C ATACAA

100 90 80 70 60

Chr03:27047054:27047156:+ 103(nt) -63.30(kcal/mol)

TCGCCATTGACTAAGCATTTCTAGTTCATAACACAGCTAAATGGGCTACACAAACATATCCATTTAGCCGTGTTATGAACTAGAAATGCTTAGTCAGTGCCAA

..((.(((((((((((((((((((((((((((((.((((((((((.............)))))))))).)))))))))))))))))))))))))))))))... (-63.30)

-----------TAAGCATTTCTAGTTCATAACA_1-------------------------------------TTATGAACTAGAAATGCTTAG_7

------------AAGCATTTCTAGTTCATAACA_2

27) cand_mir_2-5p

10 20 30 40

TA C-| C C G A CTA G

TATC GT CCAAAA GAATGCAA TCTAGC ATGCAC GACAA T

ATGG CA GGTTTT CTTACGTT AGATCG TACGTG CTGTT G

TG AA^ A T A A TAC T

90 80 70 60 50

Chr01:10598048:10598146:+ 99(nt) -42.70(kcal/mol)

TATATCCGTCCCAAAACGAATGCAAGTCTAGCAATGCACCTAGACAAGTGTTTGTCCATGTGCATAGCTAGAATTGCATTCTTTTTGGAACAAGGTAGT

..((((.((.((((((.((((((((.((((((.((((((...(((((....)))))...)))))).)))))).)))))))).)))))).))..)))).. (-42.70)

----------CCAAAACGAATGCAAGTCTAG_5------------------------------------AGAATTGCATTCTTTTTGGAA_1

**Additional file 7. Secondary structures of novel miRNA candidates with miRNA***

In the sequences above, T denotes Uracil.
