# Supplementary material for: Identification of four functionally important microRNA families with contrasting differential expression profiles between drought-tolerant and susceptible rice leaf at vegetative stage
Source: BMC Genomics. 2015 Sep 15;16(1):692. doi: 10.1186/s12864-015-1851-3 (PMC4570225; doi:10.1186/s12864-015-1851-3)
Supplement: Additional file 8: — Differentially expressed known Oryza sativa miRNAs. (A) Conserved miRNA families and (B) Non- conserved miRNA families that are differentially expressed under drought stress [82–89]. (DOCX 40 kb) [file 12864_2015_1851_MOESM8_ESM.docx]

**Additional file 8. Differentially expressed *Oryza sativa* known miRNAs**

(A) Conserved miRNA families

| miRNA families | Leaf | Stem | Targets (bolded targets = previously annotated targets) | Literature review (expression + functional information) taken mostly from references [49, 52]. |
| --- | --- | --- | --- | --- |
| osa-MIR156 | -osa-miR156a/b-5p/c-5p/e/g-5p/i/ (R↑)  -osa-miR156d/f-5p/h-5p/j-5p (R↑)  -osa-miR156k (R A↑)  -osa- miR156f-3p*/h-3p*/l-3p* (R↑) |  | **Squamosa promoter binding protein (SPL)**  **Squamosa promoter binding protein**  **Squamosa promoter binding protein**  Receptor-like protein kinase | -In our study, 11 out of 12 members of this family are induced in leaf of IR64 under drought stress.  -miR156 family was reported to be induced by salt and drought stresses in *A. thaliana* [15].  -SPL TFs play important roles in leaf development and vegetative phase change [48]. |
| osa-MIR159 | -osa-miR159a.2* (V R↑)  -osa-miR159f (V R↑) | -osa-miR159a.2* (R A↑)  -osa-miR319b (R↑) | PPR-repeat protein, Heat shock protein  **MYB family transcription factor**  **Transcription factor PCF6** | -miR159 family was reported to be induced by drought and ABA [82] and salt [15] in *A. thaliana*.  -ABA is a key plant stress hormone produced under drought stress and mediates the expression of stress-related genes and the initiation of stomatal closure. MYB are positive regulators of ABA signaling [52]. |
| osa-MIR160 | -osa-miR160a-5p/b-5p/c-5p (V A↑)  -osa-miR160d-5p/e-5p (V↑)  - osa-miR160f-5p (V↑) | -osa-miR160a-5p/b-5p/c-5p (V↓A↑)  -osa-miR160d-5p/e-5p (V↓A↑)  -osa-miR160f-5p (V↓)  -osa-miR160a-3p*/b-3p* (V R↓)  -osa-miR160c-3p* (V R↓)  -osa-miR160f-3p* (R↓) | **Auxin response factor** **(ARF)** 10 & 13  **Auxin response factor** 10 & 13  **Auxin response factor** 10 & 13  Chloride channel  Chloroplast SRP receptor cp precursor  Receptor-type protein kinase LRK1 | -In our study, 6 out of 6 members of this family are responsive to drought treatment especially in Vandana.  -ARFs are important TFs involved in auxin signal transduction by binding to specific cis-elements in the upstream regions of the auxin- inducible genes. Both miR160 and miR167 was also reported to play major roles in drought and ABA response in plants [52]. |
| osa-MIR164 |  | -osa-miR164c (A↓)  -osa-miR164e (R↓) | **NAC transcription factor**  **NAC transcription factor** | -In our study, we find that its downregulation is specific to stem.  -NAC TFs are important in root and shoot development [49]. |
| osa-MIR166 | -osa-miR166e-3p (A↓)  -osa-miR166a-5p* (R↓)  -osa-miR166h-5p* (All↓)  -osa-miR166k-5p* (All↓) | -osa-miR166e-3p (A↓)  -osa-miR166i-3p (R↓)  -osa-miR166a-5p* (R↓)  -osa-miR166b-5p* (R↓)  -osa-miR166d-5p* (R↓)  -osa-miR166h-5p* (All↓)  -osa-miR166k-5p* (V R↓) | Alkaline neutral invertase  **Homeodomain-leucine zipper protein**  Sugar transporter family  Exonuclease  Exonuclease  Diaminopimelate decarboxylase, U-box domain containing, Stress-induced protein STI1  Hypothetical protein | -miR166 was downregulated in response to drought in rice [19] but upregulated in M. Truncatula [25].  -MIR166, which targets HD-Zip, plays important roles in root and nodule development [25].  -In this study, more miRNA* of MIR166 family are found to be downregulated instead of mature miRNA. This is not surprising in view of the biological function of these mature miRNAs in root development. This study uses leaf and stem tissues instead. |
| osa-MIR167 | -osa-miR167a-5p/b/c-5p (A↑) | -osa-miR167h-3p* (All↓) | Glutamate transporter  Zinc finger, Serine Threonine kinase | -miR167 was induced by drought in *A. thaliana* [15] and was downregulated by ABA treatment in rice seedlings [83].  -It targets ARF6 and ARF8, and regulates both female and male reproduction [84].  -In maize, phospholipase D (PLD), a positive regulator of drought stress resistance, was predicted as a target of miR167d. PLD was reported to direct ABA response and affect stomatal movement in guard cells [63]. |
| osa-MIR169 | -osa-miR169i-3p* (V A↓)  osa-miR169r-3p* (V R↓) | -osa-miR169f.2 (R↓)  -osa-miR169n/o (R↓)  osa-miR169r-3p* (V R↓) | D-isomer specific 2-hydroxyacid dehydrogenase  Hypothetical protein  LRR receptor-like kinase  UDP-glucose 4-epimerase | -miR169 has been studied in detail in response to ABA and drought. For example, miR169g was induced by drought in rice [18] and miR169a was downregulated by drought stress and ABA treatment in *A. thaliana* [17].  -miR169 targets the NFYA5 TF, which controls stomatal aperture and the expression of a number of drought-responsive genes, such as glutathione transferase or peroxidise [17].  -In *M. truncatula*, miR169 was downregulated only in the roots [25]. This may explain why only a few mature miRNAs of this family are found to be differentially expressed in our study. |
| osa-MIR171 | -osa-miR171c-5p* (V↓)  -osa-miR171e-5p* (V↓) | -osa-miR171c-5p* (All↓)  -osa-miR171d-5p* (V↓)  -osa-miR171e-5p* (All↓) | Phosphatase, MRP-like ABC transporter  Serine Threonine protein kinase  Hypothetical protein | -In Arabidopsis, miR171 responded to salt, mannitol and cold treatments [15].  -The SCR family is targeted by miR171c to promote the proper development of auxiliary meristems during shoot branching in Arabidopsis [85].  -In this study, more miRNA* of MIR171 family are found to be downregulated instead of mature miRNA. |
| osa-MIR172 |  | -osa-miR172d-5p* (R↓) | F-box/ LRR-repeat MAX2 homolog | -miR172 is an important regulator of the floral patterning genes such as APETALA2, TOE1 and TOE2 [49]. Therefore, it is not surprising not to find any of the mature miRNA of this family to be differentially expressed. |
| osa-MIR390 | -osa-miR390-3p* (R↓) | -osa-miR390-5p (R↓)  -osa-miR390-3p* (R A↓) | Extra sporogenous cells, Pto kinase interactor,  Transmembrane protein kinase  Aminophospholipid transporter | -miR390 was upregulated under drought stress in cowpea [86].  -It targets TAS3, which regulates lateral root emergence and organ polarity establishment by targeting TFs such as ARF2, ARF3 and ARF4 [87]. |
| osa-MIR393 | -osa-miR393b-3p** (R↑) | -osa-miR393a (A↑) | **Transport inhibitor response 1-like protein**  Hypothetical protein | -It is known that miR393 was commonly upregulated during drought stress in Arabidopsis [16] and rice [18].  -The target of miR393 encodes TIR1, an auxin receptor in Arabidopsis. The TIR1 enzyme is a positive regulator of auxin signaling by promoting the degradation of Aux/IAA proteins through ubiquitination. The increased levels of miR393 would downregulate auxin signaling and may reduce plant growth under drought stress [52]. |
| osa-MIR394 | -osa-miR394 (V↑) | -osa-miR394 (R A↑) | RNA polymerase sigma factor | - No relevant information. |
| osa-MIR396 |  | -osa-miR396a-3p* (R↓)  -osa-miR396c-3p* (R↓)  -osa-miR396e-3p* (R↓)  -osa-miR396f-3p* (A↓) | Peroxisomal membrane protein  ATP-binding-cassette protein, germin-like protein  Avr9/Cf-9 rapidly elicited protein 141  Disease resistance protein RPM1 | -miR396 was found to be downregulated by drought in rice [19] and cowpea [86], but upregulated in drought-stressed Arabidopsis [15].  -miR396 plays an important role in plant leaf growth and development, most likely by repressing the identified target mRNAs encoding members of the GRF family of transcription factors [83]. |
| osa-MIR397 | -osa-miR397a (All ↓↑↓)  -osa-miR397b (V A↓) | -osa-miR397a (V A↓)  -osa-miR397b (V A↓) | **Laccases**, Osmotic stress-activated protein kinase  **Laccases**, Osmotic stress-activated protein kinase | -Refer text for detailed explanation for this family. |
| osa-MIR398 | -osa-miR398b (V A↓) | -osa-miR398b (V A↓) | Replication protein A1, Soluble starch synthase III-1 | -Refer text for detailed explanation for this family. |
| osa-MIR399 | -osa-miR399a/b/c (V↓)  -osa-miR399j (V↓) | -osa-miR399j (R↓) | Hypothetical protein  Hypothetical protein | -Phosphate (Pi) homeostasis is under miR399 regulation. It targets the phosphate 2 gene (PHO2), which has a role in protein degradation pathway [88]. |
| osa-MIR408 | -osa-miR408-5p (V A↓) | -osa-miR408-5p (All↓) | Leucine-rich receptor-like protein kinase | -Refer text for detailed explanation for this family. |
|  | -osa-miR408-3p* (V A↓) | -osa-miR408-3p* (V A↓) | GRAS family transcription factor containing protein, Zinc C3HC4 type family expressed |  |
| osa-MIR827 | -osa-miR827 (R↑) |  | NB-ARC domain containing protein | -It targets SPX-domain-containing genes that negatively regulate other phosphorus-responsive genes. miR399 and miR827 are involved in conserved phosphorus deficiency signaling pathways [89]. |

(B) Non- conserved miRNA families

| miRNA families | Leaf | Stem | Targets |
| --- | --- | --- | --- |
| osa-MIR1320 | -osa-miR1320-5p (V↓) |  | Ethylene-responsive element binding protein |
| osa-MIR1423 | -osa-miR1423-3p*(V↑) | -osa-miR1423-3p*(V↑) | Cytochrome P450-dependent fatty acid hydroxylase |
| osa-MIR1425 | -osa-miR1425-5p** (V R↑) | -osa-miR1425-5p** (V ↓A↑) | Leucine zipper |
| osa-MIR1429 | -osa-miR1429-5p (V↓) |  | - |
| osa-MIR1432 | -osa-miR1432-5p  (V↓) |  | Calcium-binding protein, Zinc transporter 6 |
| osa-MIR1846 |  | -osa-miR1846d-5p (A↓) | CBS domain-containing protein |
| osa-MIR1847 | -osa-miR1847.1 (V R↑) |  | Sensory histidine kinase |
| osa-MIR1848 | -osa-miR1848 (A↓) |  | RNA helicase |
| osa-MIR1849 | -osa-miR1849 (V↑) |  | Transportin-1 |
| osa-MIR1850 |  | -osa-miR1850.1 (R↓) | Protein kinase domain containing protein |
| osa-MIR1861 | -osa-miR1861a/o (V↓) | -osa-miR1861a/o (All↓↓↑) | Replication protein A 70kDa, SMC5 protein |
|  | -osa-miR1861b/f/i/l (V↓A↑) |  | SMC5 protein |
|  | -osa-miR1861d (A↑) |  | SMC5 protein, Receptor like protein kinase, BURP domain-containing protein, TCP family transcription factor containing protein |
|  |  | -osa-miR1861e/k/m (A↓) | Probable auxin efflux carrier component 2, Glutathione peroxidase 4, Mitotic control protein DIS3, Pentatricopeptide repeat-containing |
|  | -osa-miR1861g (A↓) | -osa-miR1861g (A↓) | Glutathione peroxidase 4, Mitotic control protein DIS3, Pentatricopeptide repeat-containing |
|  | -osa-miR1861h/j (A↑) | -osa-miR1861h/j (R↓) | SMC5 protein, Extracellular solute-binding family 7, Receptor like protein kinase |
| osa-MIR1862 | -osa-miR1862a/b/c (V↑) | -osa-miR1862a/b/c (V↑) | Hydrolase |
| osa-MIR1873 | -osa-miR1873 (V↑A↓) | -osa-miR1873 (V↑A↓) | Receptor kinase TrkA |
| osa-MIR1874 |  | -osa-miR1874-3p (R↓) | - |
| osa-MIR1880 |  | -osa-miR1880 (R↓) | Cytochrome c, RNA-binding protein Musashi homolog 2-like |
| osa-MIR2863 |  | -osa-miR2863b (R↓) | ATPase ATP1 |
| osa-MIR2864 | -osa-miR2864.1 (V↑) |  | Aldehyde dehydrogenase, Snf7 family protein |
|  | -osa-miR2864.2 (V↑) |  | Serine Threonine protein kinase, 101 kDa Heat shock protein |
| osa-MIR2865 |  | -osa-miR2865 (R↓) | Probable protein phosphatase 2C 66 |
| osa-MIR2871 |  | -osa-miR2871a-5p (R↓) | - |
| osa-MIR2873 | -osa-miR2873a (V↑) | -osa-miR2873a (V R↑) | Receptor-like protein kinase |
| osa-MIR2878 | -osa-miR2878-5p (V↑) | -osa-miR2878-5p (V↑) | Arm repeat-containing protein, UDP-glucose:sterol glucosyltransferase |
| osa-MIR3979 |  | -osa-miR3979-5p (R↓) | - |
|  |  | -osa-miR3979-3p** (R↓) | Translation elongation factor protein |
| osa-MIR3980 | -osa-miR3980a-3p*/b-3p* (V R↑) |  | Protein kinase Xa21 receptor type-like protein |
| osa-MIR444 |  | -osa-miR444a-3p.1/d.1 (A↑) | MADS-box transcription factor, Zinc finger protein, Chloroplastic-like Serine Threonine protein kinase |
|  |  | -osa-miR444a-3p.2/d.2 (A↑) | MADS-box transcription factor, Zinc finger protein, Chloroplastic-like Serine Threonine protein kinase |
|  |  | -osa-miR444e (A↑) | MADS-box transcription factor, Zinc finger protein, Chloroplastic-like Serine Threonine protein kinase |
|  |  | -osa-miR444f (R↓) | Zinc finger protein |
| osa-MIR5076 |  | -osa-miR5076 (R A↓) | BHLH transcription factor-like, F-box protein |
| osa-miR5144 | -osa-miR5144-3p* (R↑) | -osa-miR5144-3p* (A↓) | Zinc finger protein, Probable membrane-associated kinase regulator 4-like |
| osa-MIR5145 | -osa-miR5145 (R↓) |  | ATP-dependent Clp protease ATP-binding subunit ClpA |
| osa-MIR5146 | -osa-miR5146 (R↓) |  | - |
| osa-MIR5159 |  | -osa-miR5159 (V↑) | Prolyl carboxypeptidase like |
| osa-MIR528 | -osa-miR528-5p (V A↓) | -osa-miR528-5p (All ↓) | Laccase-2, Superoxide dismutase, Ring zinc finger protein |
|  | -osa-miR528-3p* (V A↓) | -osa-miR528-3p* (V A↓) | Serine Threonine kinase 38, Galactosyltransferase |
| osa-MIR530 | -osa-miR530-3p (V↓) | -osa-miR530-3p (R↓) | Golgi snare 12 protein |
|  | -osa-miR530-5p* (A↓) | -osa-miR530-5p* (R↓A↑) | Protein kinase AKINbetagamma-2, Rubisco activase b |
| osa-MIR531 | -osa-miR531a/c (V↓) |  | Valine-pyruvate aminotransferase 3 |
|  | -osa-miR531b (V↓) |  | Transferase family protein |
| osa-MIR5337 | -osa-miR5337b (R↓) |  | Chloroplast nucleoid DNA-binding protein |
| osa-MIR535 | -osa-miR535-3p* (V R↑) |  | Metallopeptidase family M24 containing, Protein MEI2-like 2 |
| osa-MIR5493 |  | -osa-miR5493 (A↑) | Probable NAD H-dependent oxidoreductase 2 |
| osa-MIR5504 | -osa-miR5504 (R↑) |  | Serine hydroxymethyltransferase |
| osa-MIR5795 | -osa-miR5795 (V↑) | -osa-miR5795 (V↑) | Hypothetical protein |
| osa-MIR5802 | -osa-miR5802 (V↓) | -osa-miR5802 (V R↑) | - |
| osa-MIR5803 | -osa-miR5803 (V↓) |  | - |
| osa-MIR5805 | -osa-miR5805 (V↑) | -osa-miR5805 (V↑) | - |
| osa-MIR5807 | -osa-miR5807 (V↑) | -osa-miR5807 (V↑) | Ethylene-forming enzyme |
| osa-MIR5821 | -osa-miR5821 (V↓) |  | Endonuclease/exonuclease/phosphatase, Embryogenesis transmembrane protein |
| osa-MIR6248 |  | -osa-miR6248 (R↓) | 60s Ribosomal protein L1, BHLH transcription factor-like, DNA-directed RNA polymerase III subunit rpc1-like, Probable auxin efflux carrier component 2 |
| osa-MIR6249 |  | -osa-miR6249a/b (A↑) | 9-cis-epoxycarotenoid dioxygenase |
| osa-MIR6253 | -osa-miR6253 (V A↓) | -osa-miR6253 (A↑) | - |
| osa-MIR6254 |  | -osa-miR6254 (R↓) | Apoptosis-related RNA binding protein |
| osa-MIR810 | -osa-miR810b.1 (V R↑) |  | Pentatricopeptide repeat-containing protein |
|  | -osa-miR810b.2 (V R↑) | -osa-miR810b.2 (V↑) | DNA polymerase alpha catalytic subunit |
| osa-MIR812 | -osa-miR812k/l/m (R↓) | -osa-miR812k/l/m (V↑) | - |
|  | -osa-miR812p (A↓) |  | E1-E2 ATPase family protein |
| osa-MIR815 |  | -osa-miR815a (R↓) | Importin-beta N-terminal domain containing, Membrane-associated salt-inducible protein like, Probable protein phosphatase 2C 45 |
| osa-MIR818 | -osa-miR818d (R↓) |  | ABC transporter-like protein |

V=Vandana, A=Aday Sel and R=IR64. The arrows indicating up-regulation or down-regulation are arranged in the order of Vandana, IR64 and Aday Sel.
