# Supplementary material for: Identification of four functionally important microRNA families with contrasting differential expression profiles between drought-tolerant and susceptible rice leaf at vegetative stage
Source: BMC Genomics. 2015 Sep 15;16(1):692. doi: 10.1186/s12864-015-1851-3 (PMC4570225; doi:10.1186/s12864-015-1851-3)
Supplement: Additional file 9: — Enrichment of GO biological processes in leaf. (DOCX 20 kb) [file 12864_2015_1851_MOESM9_ESM.docx]

osa-miR2864.1 ; Snf 7 family protein ; V↑

Protein transport

osa-miR169r-3p* ; UDP-glucose 4-epimerase ; V↓R↓

osa-miR166e-3p ; Alkaline neutral invertase ; A↓

Root development

osa-miR397a/osa-miR397b ; Osmotic stress activated protein kinase ; V↓R↑A↓

osa-miR5504 ; serine hydroxymethyltransferase; R↑

osa-miR166h-5p* ; Diaminopimelate decarboxylase ; All↓

osa-miR166e-3p ; Alkaline neutral invertase ; A↓

osa-miR2878-5p ; UDP-glucose:sterol glucosyltransferase ; V↑

osa-miR169r-3p* ; UDP-glucose 4-epimerase ; V↓R↓

Lignin catabolic process

osa-miR397a/osa-miR397b ; Laccase lac 5-4 ; V↓R↑A↓

osa-miR397a/osa-miR397b ; Laccase-22 ; V↓R↑A↓

Cellular amino acid metabolic process

Carbohydrate metabolic process

osa- miR1861g ; Glutathione peroxidase 4 ; A↓

osa-miR530-5p* ; Protein kinase AKINbetagamma-2 ; A↓

osa-miR169r-3p* ; UDP-glucose 4-epimerase ; V↓R↓

Response to salt stress

osa-miR1432-5p ; Zinc transporter 6 ; V↓

osa-miR167a-5p/osa-miR167b/osa-miR167c-5p ; Glutamate transporter ; A↑

osa-miR390-3p* ; Aminophospholipid transporter ; R↓

Transport

Cation transport

Transmembrane transport

osa-miR530-3p ; Golgi SNARE 12 proten ; V↓

Response to stress

osa-miR1847.1 ; Sensor histidine kinase ; V↑R↑

osa-miR166h-5p* ; U-box domain containing protein ; All↓

osa-miR528-3p* ; Serine/Threonine kinase 38 ; V↓A↓

osa-miR2864.2 ; Serine/Threonine protein kinase ; V↑

Protein phosphorylation

osa-miR2873a ; Receptor-like protein kinase ; V↑

osa-miR397a/osa-miR397b ; Osmotic stress-activated protein kinase ; V↓R↑A↓

Protein metabolic process

Protein ubiquitination

osa-miR166h-5p* ; Stress-induced protein STI1 ; All↓

osa-miR2878-5p ; ARM repeat-containing protein ; V↑

Oxidation-reduction process

osa-miR397a/osa-miR397b ; Laccase-22 ; V↓R↑A↓

osa-miR2864.1 ; Aldehyde dehydrogenase ; V↑

osa- miR1861g ; Glutathione peroxidase 4 ; A↓

DNA replication

osa-miR398b ; Replication protein A1 ; V↓A↓

osa-miR1861a/osa-miR1861o ; Replication protein A 70kDa ; V↓

osa-miR810b.2 ; DNA polymerase alpha catalytic subunit ; V↑R↑

osa-miR1423-3p* ; Cytochrome P450-dependent fatty acid hydroxylase ; V↑

osa-miR397a/osa-miR397b ; Laccase lac 5-4 ; V↓R↑A↓

**Additional file 9. Enrichment of GO biological processes in leaf**
