# Supplementary material for: Identification of four functionally important microRNA families with contrasting differential expression profiles between drought-tolerant and susceptible rice leaf at vegetative stage
Source: BMC Genomics. 2015 Sep 15;16(1):692. doi: 10.1186/s12864-015-1851-3 (PMC4570225; doi:10.1186/s12864-015-1851-3)
Supplement: Additional file 10: — Enrichment of GO biological processes in stem. (DOCX 23 kb) [file 12864_2015_1851_MOESM10_ESM.docx]

osa-miR397a/osa-miR397b ; Laccase lac 5-4 ; V↓A↓

osa-miR397a/osa-miR397b ; Laccase-22 ; V↓A↓

Lignin catabolic process

osa-miR393a ; Transport inhibitor response 1-like protein ; A↑

osa-miR530-5p* ; Protein kinase AKINbetagamma-2 ; R↓A↑

osa-miR169r-3p* ; UDP-glucose 4-epimerase ; V↓R↓

osa- miR1861e/k/m/g ; Glutathione peroxidase 4 ; A↓

Response to stress

Response to oxidative stress

osa-miR2871a-5p ; Hypothetical protein OsI_04842 ; R↓

Response to salt stress

osa-miR397a/osa-miR397b ; Osmotic stress-activated protein kinase ; V**↓**A**↓**

Response to osmotic stress

osa-miR1861h/osa-miR1861j ; Extracellular solute-binding family 7 ; R↓

Transport

osa-miR1425-5p** ; Leucine zipper ; V↓A↑

osa-miR396c-3p* ; ATP-binding-cassette protein ; R↓

osa-miR390-3p* ; Aminophospholipid transporter ; R↓A↓

osa-miR530-3p ; Golgi SNARE 12 proten ; R↓

osa-miR815a ; Importin-beta N-terminal domain containing,expressed ; R↓

osa-miR390-5p ; Extra sporogenous cells ; R↓

Intracellular protein transport

Protein transport

Stamen development

Root development

osa-miR393a ; Transport inhibitor response 1-like protein ; A↑

osa-miR169r-3p* ; UDP-glucose 4-epimerase ; V↓R↓

osa-miR166e-3p ; Alkaline neutral invertase ; A↓

Embryo development ending in seed dormancy

osa- miR1861e/k/m/g ; Glutathione peroxidase 4 ; A↓

osa-miR166i-3p ; Homeobox-leucine zipper protein HOX32 ; R↓

Cell Wall biogenesis

osa-miR2863b ; atp1-ATPase ; R↓

osa-miR169r-3p* ; UDP-glucose 4-epimerase ; V↓R↓

osa-miR160c-3p* ; Chloroplast SRP receptor cp precursor; V↓R↓

DNA replication

DNA replication

osa-miR810b.2 ; DNA polymerase alpha catalytic subunit ; V↑

osa-miR1861a/o ; Replication protein A 70kDa ; V↓R↓A↑

osa-miR398b ; Replication protein A1 ; V↓A↓

Oxidation-reduction process

osa-miR397a/osa-miR397b ; Laccase-22 ; V↓A↓

osa-miR397a/osa-miR397b ; Laccase lac 5-4 ; V↓A↓

osa-miR1423-3p* ; Cytochrome P450-dependent fatty acid hydroxylase ; V↑

osa-miR169f.2 ; D-isomer specific 2-hydroxyacid dehydrogenase ; R↓

osa- miR1861e/k/m/g ; Glutathione peroxidase 4 ; A↓

osa-miR5076 ; E3 ubiquitin-protein ligase UPL4-like ; R↓A↓

osa-miR815a ; Probable protein phosphatase 2C 45 ; R↓

Protein ubiquitination

osa-miR2878-5p ; ARM repeat-containing protein ; V↑

Cellular protein modification process

Protein phosphorylation

osa-miR166h-5p* ; U-box domain containing protein ; All↓

osa-miR171d-5p* ; Serine/Threonine protein kinase ; V↓

osa-miR2873a ; Receptor-like protein kinase ; V↑R↑

osa-miR397a/osa-miR397b ; Osmotic stress-activated protein kinase ; V↓A↓

osa-miR528-3p* ; Serine/Threonine kinase 38 ; V↓A↓

osa-miR390-5p ; Extra sporogenous cells ; R↓

osa-miR390-5p ; Pto kinase interactor ; R↓

osa-miR390-5p ; Transmembrane protein kinase ; R↓

osa-miR444a-3p.1/d.1/a-3p.2/d.2/e ; Serine/Threonine protein kinase, chloroplastic-like ; A↑

osa-miR166h-5p* ; Stress-induced protein STI1 ; All↓

Cellular amino acid metabolic process

osa-miR166h-5p* ; Diaminopimelate decarboxylase ; All**↓**

Carbohydrate metabolic process

osa-miR166e-3p ; Alkaline neutral invertase ; A↓

osa-miR2878-5p ; UDP-glucose:sterol glucosyltransferase ; V↑

osa-miR169r-3p* ; UDP-glucose 4-epimerase ; V↓R↓

Transcription, DNA-dependent

osa-miR166i-3p ; Homeobox-leucine zipper protein HOX32 ; R↓

osa-miR444f ; B1358B12.3 protein ; R↓

osa-miR6248 ; DNA-directed RNA polymerase III subunit RPC-1-like ; R↓

osa-miR444a-3p.1/d.1/a-3p.2/d.2/e ; B1358B12.3 protein ; A↑

**Additional file 10. Enrichment of GO biological processes in stem**
